# Treatment Disparities in Radiation and Hormone Therapy Among Women Covered by Medicaid vs Private Insurance in Cancer Registry and Claims Data

**DOI:** 10.1001/jamahealthforum.2023.0673

**Published:** 2023-05-05

**Authors:** Cathy J. Bradley, Lindsay M. Sabik, Rifei Liang, Richard C. Lindrooth, Marcelo C. Perraillon

**Affiliations:** 1 University of Colorado Cancer Center, Aurora; 2 Colorado School of Public Health, Aurora; 3 University of Pittsburgh Graduate School of Public Health, Pittsburgh, Pennsylvania

## Abstract

**Importance:**

Prior research has reported undertreatment among patients with cancer who are insured by Medicaid, but this finding may be due, in part, to incomplete data in cancer registries.

**Objective:**

To compare disparities in radiation and hormone therapy between women with breast cancer covered by Medicaid and those with private insurance using the Colorado Central Cancer Registry (CCCR) and CCCR data supplemented with All Payer Claims Data (APCD).

**Design, Setting, and Participants:**

This observational cohort study included women aged 21 to 63 years who received breast cancer surgery. We linked the CCCR and Colorado APCD to identify Medicaid and privately insured women who were newly diagnosed with invasive, nonmetastatic breast cancer between January 1, 2012, and December 31, 2017. In the radiation treatment analysis, we narrowed the sample to women who received breast-conserving surgery (Medicaid, n = 1408; private, n = 1984) and in the hormone therapy analysis, we selected women who were hormone-receptor positive (Medicaid, n = 1156; private, n = 1667).

**Main Outcomes and Measures:**

We used logistic regression to estimate the likelihood of treatment within 12 months to assess whether the results varied between data sources.

**Results:**

There were 3392 and 2823 participants in the radiation and hormone therapy cohorts, respectively. The mean (SD) age was 51.71 (8.30) years in the radiation therapy cohort, and 52.00 (8.16) years in the hormone therapy cohort. Among the participants, there were 140 (4%) and 105 (4%) who were Black non-Hispanic, 499 (15%) and 406 (14%) who were Hispanic, 2602 (77%) and 2190 (78%) were White, and 151 (4%) and 122 (4%) were other/unknown in the radiation and hormone therapy cohorts, respectively. A higher percentage of women were aged 50 years or younger in the Medicaid samples (40% vs 34% in the privately insured sample) and identified as non-Hispanic Black (about 7%) or Hispanic (approximately 24%). Treatment was underreported in both sources, but to a lesser extent in the APCD (2.5% and 2.0% for Medicaid and private insurance, respectively) compared with CCCR (19.5% and 13.3% for Medicaid and private insurance, respectively). Using CCCR data, Women with Medicaid insurance were 4 (95% CI, −8 to −1; *P* = .02) and 10 (95% CI, −14 to −6; *P* < .001) percentage points less likely to have a record of radiation and hormone therapy compared with privately insured women, respectively. Using combined CCCR and APCD, no statistically significant disparity was observed in radiation or hormone therapy between Medicaid-insured and privately insured women.

**Conclusions and Relevance:**

Among women with breast cancer covered by Medicaid vs private insurance, cancer treatment disparities may be overestimated if based solely on cancer registry data.

## Key Points

**Question:**

Are documented treatment disparities between women covered by Medicaid vs private insurance associated with limitations in the recording of treatment information by cancer registries?

**Findings:**

Using combined Colorado Central Cancer Registry and All Payer Claims Database data, including 6215 patients who received breast cancer surgery, no statistically significant disparity was observed in receipt of radiation or hormone therapy between women with breast cancer covered by Medicaid and those covered by private insurance.

**Meaning:**

Researchers and policy makers may overestimate cancer treatment disparities between patients covered by Medicaid and private insurance if they rely solely on cancer registry data.

## Teaser

This cohort study compares disparities in radiation and hormone therapy between women with breast cancer covered by Medicaid and those with private insurance using the Colorado Central Cancer Registry and data supplemented with All Payer Claims Data.

## Introduction

Health insurance is among the strongest determinants of health care access and utilization.^1,2^ Access to care is especially important for patients diagnosed with cancer. For example, women diagnosed with breast cancer who are uninsured have the highest mortality rate compared with women with other insurance because they are diagnosed with late-stage disease more often and do not receive guideline-concordant care.^3,4,5^ Women insured with Medicaid also have lower cancer screening rates, higher likelihood of late-stage diagnoses, and poorer survival compared with women who are privately insured.^4,6,7^ Studies that use state central cancer registries linked with either Medicaid claims or hospital discharge data report that women with Medicaid are more likely to experience delays in receiving treatment^6,8,9,10,11^ and are less likely to be prescribed hormone therapy or radiation treatment postsurgery than privately insured women.^12^ In a North Carolina study,^13^ 1 out of 3 women insured by Medicaid did not receive radiation therapy after breast-conserving surgery.

Medicaid is the largest insurance program in the US and continues to expand in many states.^14^ Thus, understanding the sources of disparate care and outcomes to inform policy and health care delivery approaches that effectively address, and not exacerbate, health inequities is critically important. In addition, Black and Hispanic women are disproportionately enrolled in Medicaid, highlighting its role in racial and ethnic disparities in cancer outcomes.^15,16^ Medicaid is particularly relevant for breast cancer care because women who are diagnosed through the National Breast and Cervical Cancer Early Detection Program (NBCCEDP) are enrolled in Medicaid following diagnosis to cover their cancer treatment. One study^17^ reported that women enrolled in Medicaid prior to diagnosis tended to have earlier-stage cancer than women, including those covered through NBCCEDP, who enrolled in Medicaid the month of or following diagnosis, suggesting that poor outcomes may be partially due to uninsurance prior to Medicaid coverage rather than barriers inherent to Medicaid. Nonetheless, the conclusion that women with Medicaid are not given adequate care, even when accounting for enrollment timing,^18^ persists.

Although disparities in treatment between those with Medicaid and private insurance can be due to patient, clinician, and insurance factors, another possibility is that cancer registries may have incomplete treatment data, disproportionately so for patients insured with Medicaid.^19,20^ For example, population-based studies that used cancer registries in California and New York reported that women with Medicaid were less likely to receive recommended treatments, with the exception of chemotherapy.^18^ Similar treatment disparities based on registries, including the Surveillance, Epidemiology, and End Results (SEER) registry, without claims data, are reported elsewhere.^21,22^ In contrast, disparities were not observed in a Georgia study^23^ that used registry data to identify women diagnosed with breast cancer and medical records to identify the treatment they received. Building on this literature, we hypothesize that documented treatment disparities could be associated with limitations in recording treatment information by cancer registries. To test this hypothesis, we compared cancer registry reported receipt of radiation and hormone therapy among Medicaid and privately insured women diagnosed with breast cancer to radiation and hormone therapy in claims data from an All-Payer Claims Database (APCD). We provide a nuanced assessment of disparities when using cancer registry data alone compared with registry data supplemented with claims.

## Methods

### Data Sources

We used the linked Colorado APCD and Colorado Central Cancer Registry (CCCR) data, which comprised adults aged 21 years and older diagnosed with cancer in the CCCR and matched to APCD member information using Match*Pro. The CCCR is gold certified by the North American Association of Central Cancer Registries (NAACCR) and includes all residents with cancer, regardless of whether they were diagnosed in Colorado.^24^ The Colorado APCD was launched in 2012 and is administered by the Center for Improving Value in Health Care (CIVHC). The APCD includes claims from 36 commercial payers, Medicare, and Medicaid.^25^ It captures over 70% of covered lives, including about 50% of those covered by self-insured plans.^26^ The APCD does not contain claims for the Veterans Administration, Tricare, Indian Health Services, most Employee Retirement Income Security Act (ERISA)-covered employers, or uninsured or self-pay claims.^26^ Approximately one-third of private payers in Colorado are covered by ERISA, and ERISA plans are most common among large employers. The linkage rate between all patients diagnosed with a first cancer in the CCCR between January 1, 2012, through December 31, 2017, and the APCD was 93%. Assessments of validity concluded that the linked data were of high quality^27^ and APCD data on treatment and insurance status were reliable.^20^

### Cohort Selection

We selected women ages 21 to 63 years who were diagnosed with SEER summary stage local or regional breast cancer and enrolled in Medicaid or private insurance in the APCD enrollment file. According to the National Comprehensive Cancer Network^28^ (NCCN), these women were eligible for radiation therapy if they had breast-conserving surgery. They were eligible for hormone therapy if they were hormone receptor positive. We included women who received surgical treatment for breast cancer and who were continuously enrolled in either Medicaid or private insurance until death or 9 months following the month of the last surgery that occurred within 3 months following diagnosis. We allowed for multiple surgeries because some women have a lumpectomy followed by additional surgeries; radiation and hormone therapy are generally initiated following the last surgery. All codes used are provided in the eAppendix in Supplement 1. We excluded women enrolled simultaneously in Medicare and Medicaid or Medicaid and private insurance. We further limited the radiation therapy analytic sample to women who had breast-conserving surgery, resulting in 1408 and 1984 Medicaid and privately insured women, respectively. For the hormone therapy analytic sample, we selected women who had any surgery and who were estrogen receptor (ER) or progesterone receptor (PR) positive, yielding an analytical sample of 1245 and 1802 Medicaid and privately insured women, respectively. eFigures 1 and 2 in Supplement 1 summarize how the samples were derived. The Strengthening the Reporting of Observational Studies in Epidemiology (STROBE) reporting guidelines were used to ensure the reporting of this observational study.^29^

### Radiation and Hormone Therapy

We identified relevant codes for treatment in the CCCR and APCD for radiation and hormone therapy through literature reviews, SEER*Rx,^30^ Revenue Codes, Current Procedural Terminology codes, Healthcare Common Procedure Coding System codes, Diagnosis Revenue Group codes, the *International Classification of Diseases, Ninth Revision (ICD-9) and Tenth Revision (ICD-10) * codes, and National Drug Codes (eAppendix in Supplement 1).

### Covariates

We controlled for race and ethnicity (Hispanic, non-Hispanic Black, White, and other or unknown), patient age (<50, 50-63 years), year of diagnosis (2012-2017), and rurality of patient residence based on the 2010 Rural-Urban Commuting Area (RUCA) designations.^31^ The CCCR was the source of race and ethnicity and is based on patient self-report. We controlled for SEER summary stage (local or regional) and registry reporting source (hospital or outpatient). Registry reporting source reflects the source for the incident case. Because most diagnoses are determined in the hospital setting based on a pathology report, there is little variation in reporting source. In a sensitivity analysis, we linked clinicians’ zip codes to RUCA codes where possible and included clinician rurality in estimations. We also included poverty quantiles, based on patient census tract information, as a covariate in the sensitivity analysis. We did not include these in the main analyses due to missing census tract data and correlation of poverty with Medicaid insurance (eAppendix in Supplement 1).

### Statistical Analysis

We compared descriptive characteristics between Medicaid and privately insured women using χ^2^ tests or *t* tests. We report the average time to last surgery from diagnosis. We used a follow-up time of 9 months following the month of last surgery as the primary observation period; more than 93% of surgeries occurred within 3 months of diagnosis, resulting in a total follow-up time of 12 months from diagnosis. We estimated multivariable logistic regressions to predict the likelihood of records indicating receipt of radiation or hormone therapy using different combinations of treatment data sources: CCCR alone, APCD alone, or APCD and CCCR. In a sensitivity analysis, we estimated similar logistic regressions for longer observation windows (6 months to last surgery and 12 months of follow-up) because 1 possible reason for disparities is delay in treatment initiation that could affect when (and if) treatment is recorded in cancer registry data. For ease of interpretation, we reported marginal effects, which are interpreted as average differences in the probability of receiving treatment between Medicaid and privately insured patients.^32^ Stata (version 17, Stata Corp) was used to analyze the data. *P* values were 2-sided with *P* ≤  .05 considered statistically significant. This study was reviewed and deemed exempt by the Colorado multiple institutional review board. The data were deidentified. Therefore, the requirement for written informed consent was waived.

## Results

Descriptive statistics for Medicaid and privately insured women are reported in Table 1. Treatment was underreported in both sources, but to a lesser extent in the APCD (2.5% and 2.0% for Medicaid and private insurance, respectively, compared with CCCR 19.5% and 13.3% for Medicaid and private insurance, respectively). A higher percentage of women were younger than age 50 in the Medicaid samples (40% compared with 34% in the privately insured sample). Likewise, a higher percentage of women with Medicaid identified as Hispanic (approximately 24%) and non-Hispanic Black (about 7%). A slightly higher percentage of women covered by private insurance lived in urban areas (84% compared with 81%). More women in the Medicaid sample were diagnosed with regional stage disease (about 41% compared with 32% in the privately insured sample). No statistically significant difference was observed in time to last surgery, which on average, occurred within a month of diagnosis. The APCD was more likely than the CCCR to report both radiation and hormone therapy, and disparities in the receipt of treatment between women covered by Medicaid and private insurance were observed when using the CCCR alone.

**Table 1.** Characteristics of Women Eligible for Radiation or Hormone Therapy Within 9 Months Following Breast Cancer Surgery, CCCR-APCD, 2012 to 2017

Characteristic	Eligible for radiation therapy, No. (%)^a^			Eligible for hormone therapy, No. (%)^a^		
	Medicaid (n = 1408)	Private (n = 1984)	*P* value	Medicaid (n = 1156)	Private (n = 1667)	*P* value
Under reporting						
CCCR, not APCD	35 (2.5)	39 (2.0)	<.0001	27 (2.3)	55 (3.3)	<.001
APCD, not CCCR	274 (19.5)	264 (13.3)		439 (38.00)	442 (26.5)	
Age category, y						
<50	559 (39.7)	666 (33.6)	<.001	443 (38.3)	541 (32.5)	.001
50-63	849 (60.3)	1318 (66.4)		713 (61.7)	1126 (67.5)	
Race and ethnicity category						
Black non-Hispanic	101 (7.2)	39 (2.0)	<.001	79 (6.8)	26 (1.6)	<.001
Hispanic	337 (23.9)	162 (8.2)		275 (23.8)	131 (7.9)	
White	886 (62.9)	1716 (86.5)		737 (63.8)	1453 (87.2)	
Other/unknown^b^	84 (6.0)	67 (3.4)		65 (5.6)	57 (3.4)	
Rural residency^c^						
No	1128 (80.1)	1675 (84.4)	.002	922 (79.8)	1398 (83.9)	.009
Yes	178 (12.6)	212 (10.7)		152 (13.1)	189 (11.3)	
Missing	102 (7.2)	97 (4.9)		82 (7.1)	80 (4.8)	
SEER summary stage						
Localized	833 (59.2)	1354 (68.2)	<.001	673 (58.2)	1153 (69.2)	<.001
Regional	575 (40.8)	630 (31.8)		483 (41.8)	514 (30.8)	
Reporting source^d^						
Inpatient or hospital	1392 (98.9)	1970 (99.3)	.19	1146 (99.1)	1655 (99.3)	.67
Outpatient	16 (1.1)	14 (0.7)		10 (0.9)	12 (0.7)	
Radiation treatment within 9 mo following last surgery						
CCCR radiation	673 (47.8)	1041 (52.5)	.007	NA	NA	NA
APCD radiation	912 (64.8)	1266 (63.8)	.57	NA	NA	
Hormone treatment within 9 mo following last surgery						
CCCR hormone treatment	NA	NA	NA	658 (56.9)	1152 (69.1)	<.001
APCD hormone treatment	NA	NA		1070 (92.6)	1539 (92.3)	.81
Months between diagnosis and last surgery, mean (SD)^e^						
Either APCD or CCCR	1.08 (0.87)	1.16 (0.79)	.16	1.24 (0.89)	1.25 (0.79)	.80

Abbreviations: APCD, All Payer Claims Database; CCCR, Colorado Central Cancer Registry; NA, not applicable; SEER, Surveillance, Epidemiology, and End Results.

^a^ All women included had Medicaid or Private enrollment during the month of last surgery. Statistical significance between Medicaid and privately insured samples determined by χ^2^ test. Surgery for hormone therapy analysis was defined as biopsy (except fine needle biopsy), mastectomy, breast-conserving surgery, lumpectomy, lymphadenectomy, and sentinel lymphadenectomy. Surgery for radiation therapy analysis was defined as biopsy (except fine needle biopsies), breast-conserving surgery, lumpectomy, lymphadenectomy, and sentinel lymphadenectomy.

^b^ Other race and ethnicity comprises American Indian or Alaska Native, Asian and Pacific Islander, or unknown if Hispanic.

^c^ Rural residency was determined by Rural-Urban Commuting Area (RUCA) codes. RUCA codes classify census tracts using measures of population density, urbanization, and daily commuting flows. The RUCA secondary codes 1.0, 1.1, 2.0, 2.1, 3.0, 4.1, 5.1, 7.1, 8.1, and 10.1 are classified as urban commuting areas, and all other nonmissing codes are classified as not an urban commuting area.

^d^ Reporting source is the reporting source for the incidence case, not the treatment provided, which was most likely in an outpatient setting.

^e^ We applied 2 sample *t*-tests (2-tailed) with equal variances to compare the means between Medicaid and private insurance samples.

The Figure, A and B shows the relative contribution of the CCCR and APCD in reporting radiation and hormone therapy for Medicaid and privately insured women. The CCCR identified less than 3% of women who did not have evidence of radiation or hormone therapy in the APCD. The APCD identified 13% and 20%, respectively, of women covered by private and Medicaid insurance who received radiation therapy that was not reported in the CCCR. The 2 sources agreed on 50.5% and 44.3% of privately and Medicaid insured women who received radiation therapy (Figure, A). The 2 sources also agreed on 65.8% and 54.6% of women covered by private and Medicaid insurance, respectively, who received hormone therapy (Figure, B). However, the APCD identified 38% of Medicaid insured women who received hormone therapy but did not have a record of hormone therapy in the CCCR.

**Figure.**
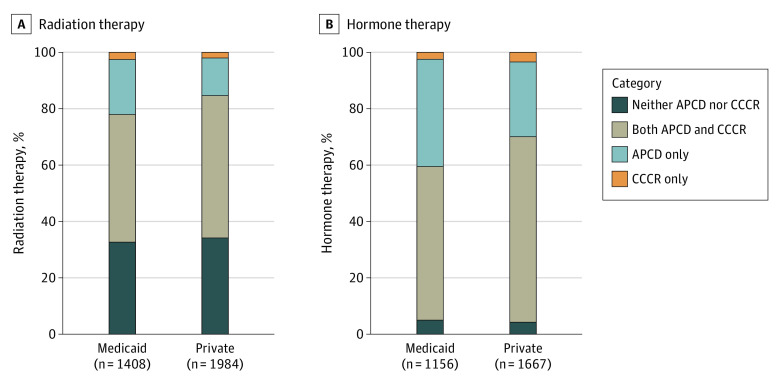
Proportion of Women Diagnosed With Breast Cancer Who Received Treatment Within 9 Months Following Last Surgery According to the CCCR and APCD, Medicaid and Private Insurance, 2012 to 2017 Abbreviations: APCD, All Payer Claims Database; CCCR, Colorado Central Cancer Registry.

Table 2 reports the marginal effects for the likelihood of radiation therapy based solely on information in the CCCR and APCD, respectively, and based on combined information from these 2 data sources. Using the CCCR alone, Medicaid insured women were 4 (95% CI,−8 to −1) percentage points less likely to receive radiation therapy than privately insured women (*P* = .02). In contrast, no statistically significant differences were observed between women covered by Medicaid and private insurance when APCD claims were used (Table 2, columns 2 and 3). Older women and women diagnosed with regional-stage disease were more likely to have a record of radiation therapy across data sources.

**Table 2.** Likelihood of Radiation Therapy Within 9 Months Following Last Surgery Depending on Information Source, CCCR-APCD, 2012 to 2017, Among 3392 Participants

Variable^a^	CCCR only, 9-mo^b^		APCD only, 9-mo^b^		Either APCD or CCCR	
	ME (95% CI)	*P* value	ME (95% CI)	*P* value	ME (95% CI)	*P* value
Insurance						
Private	1 [Reference]	.02	1 [Reference]	.95	1 [Reference]	.93
Medicaid	−0.04 (−0.08 to −0.01)		0 (−0.03 to 0.04)		0 (−0.03 to 0.03)	
Age category, y						
<50	1 [Reference]	<.001	1 [Reference]	<.001	1 [Reference]	<.001
50-63	0.16 (0.13 to 0.2)		0.11 (0.07 to 0.14)		0.12 (0.09 to 0.15)	
Race and ethnicity category						
Black non-Hispanic	−0.02 (−0.10 to 0.07)	.67	−0.01 (−0.10 to 0.07)	.75	0.01 (−0.07 to 0.09)	.80
Hispanic	0.01 (−0.03 to 0.06)	.56	0.00 (−0.05 to 0.05)	.99	0.01 (−0.03 to 0.06)	.61
White	1 [Reference]	NA	1 [Reference]	NA	1 [Reference]	NA
Other/unknown	0.04 (−0.04 to 0.12)	.30	0.02 (−0.06 to 0.10)	.59	0.03 (−0.05 to 0.10)	.49
Rural residency						
No	1 [Reference]	NA	1 [Reference]	NA	1 [Reference]	NA
Yes	−0.04 (−0.09 to 0.01)	.12	−0.02 (−0.07 to 0.03)	.45	−0.03 (−0.08 to 0.03)	.32
Missing	0 (−0.07 to 0.07)	.92	0.01 (−0.06 to 0.08)	.79	0.01 (−0.06 to 0.07)	.84
SEER summary stage						
Localized	1 [Reference]	.001	1 [Reference]	<.001	1 [Reference]	<.001
Regional	0.06 (0.02 to 0.09)		0.18 (0.15 to 0.21)		0.19 (0.16 to 0.22)	
Reporting source						
Inpatient or hospital	1 [Reference]	.84	1 [Reference]	.33	1 [Reference]	.23
Outpatient	0.02 (−0.16 to 0.19)		0.08 (−0.08 to 0.24)		0.09 (−0.06 to 0.25)	

Abbreviations: APCD, All Payer Claims Database; CCCR, Colorado Central Cancer Registry; ME, marginal effect; NA, not applicable; SEER, Surveillance, Epidemiology, and End Results.

^a^ All women were continuously enrolled in Medicaid or an APCD plan. Year of diagnosis included, but coefficients are not reported. Footnotes to Table 1 apply.

^b^ Radiation treatment was defined as patients who received radiation therapy within 9 months following breast cancer surgery (biopsy [except fine needle biopsy], breast-conserving surgery, lumpectomy, lymphadenectomy, and sentinel lymphadenectomy included) from CCCR data or APCD claims data.

Table 3 reports the marginal effects for hormone therapy, depending on data source. Women with Medicaid insurance were 10 (95% CI, −14 to −6) percentage points less likely to have a record of hormone therapy compared with privately insured women when using the CCCR alone (*P* < .001). When we used the APCD, either alone or pooled with the CCCR, there was no statistical difference in hormone therapy between women covered by Medicaid and private insurance. The disparity between rural and urban residents was also no longer statistically significant.

**Table 3.** Likelihood of Hormone Therapy Within 9 Months Following Last Surgery Depending on Information Source, CCCR-APCD, 2012 to 2017, Among 2823 Participants

Variable^a^	CCCR only, 9 mo^b^		APCD only, 9 mo^b^		Either APCD or CCCR	
	ME (95% CI)^b^	*P* value	ME (95% CI)^b^	*P* value	ME (95% CI)^b^	*P* value
Insurance						
Private	1 [Reference]	<.001	1 [Reference]	.51	1 [Reference]	.19
Medicaid	−0.10 (−0.14 to −0.06)		−0.01 (−0.03 to 0.01)		−0.01 (−0.03 to 0.01)	
Age category, y						
<50	1 [Reference]	.009	1 [Reference]	.41	1 [Reference]	.39
50-63	0.05 (0.01 to 0.08)		−0.01 (−0.03 to 0.01)		0.01 (−0.01 to 0.02)	
Race and ethnicity category						
Black non-Hispanic	0.02 (−0.07 to 0.11)	.71	0.01 (−0.04 to 0.06)	.70	−0.01 (−0.05 to 0.04)	.80
Hispanic	0.01 (−0.04 to 0.06)	.77	0.01 (−0.01 to 0.04)	.28	0.02 (0.00 to 0.04)	.04
White	1 [Reference]	NA	1 [Reference]	NA	1 [Reference]	NA
Other/unknown	−0.01 (−0.09 to 0.08)	.90	0.02 (−0.03 to 0.06)	.45	0.01 (−0.03 to 0.05)	.73
Rural residency						
No	1 [Reference]	NA	1 [Reference]	NA	1 [Reference]	NA
Yes	−0.11 (−0.16 to −0.05)	<.001	−0.01 (−0.04 to 0.02)	.56	−0.02 (−0.04 to 0.01)	.25
Missing	−0.05 (−0.12 to 0.03)	.21	0 (−0.04 to 0.04)	.91	0 (−0.04 to 0.03)	.85
SEER summary stage						
Localized	1 [Reference]	<.001	1 [Reference]	.001	1 [Reference]	.13
Regional	−0.12 (−0.16 to −0.09)		0.03 (0.01 to 0.05)		0.01 (−0.00 to 0.03)	
Reporting source						
Inpatient or hospital	1 [Reference]	.72	1 [Reference]	.28	1 [Reference]	.63
Outpatient	0.03 (−0.15 to 0.22)		−0.08 (−0.22 to 0.07)		−0.02 (−0.12 to 0.07)	

Abbreviations: APCD, All Payer Claims Database; CCCR, Colorado Central Cancer Registry; ME, marginal effect; NA, not applicable; SEER, Surveillance, Epidemiology, and End Results.

^a^ All women are continuously enrolled in Medicaid or an APCD plan. Year of diagnosis included, but coefficients are not reported. Footnotes to Table 1 apply.

^b^ Hormone therapy was defined as patients who received hormone therapy within 9 months following breast cancer surgery (biopsy [except fine needle biopsy], mastectomy, breast-conserving surgery, lumpectomy, lymphadenectomy, and sentinel lymphadenectomy included) from CCCR data or APCD claims data.

In a sensitivity analysis, we increased the follow-up time to 12 months following the month of last surgery (eTables 1 and 2 in Supplement 1). Again, no statistically significant differences in the receipt of radiation and hormone therapy were observed between women covered by Medicaid and private insurance when APCD claims were used. We added poverty quantiles and a variable for whether the clinician was in a rural area (eTables 3 and 4 in Supplement 1). Poverty was not statistically significantly associated with radiation or hormone treatment underreporting. The coefficient for Medicaid, however, became smaller and less statistically significant for radiation treatment in the CCCR-only analyses. The CCCR was 3 percentage points (95% CI, −7 to 0) less likely to report radiation therapy for women covered by Medicaid relative to privately insured women. Rural clinicians were 7 percentage points (95% CI, 0-14) more likely to report radiation treatment to the APCD than urban clinicians. Findings were similar for hormone treatment.

## Discussion

For women who have breast-conserving surgery, radiation therapy is effective in decreasing mortality and recurrence.^12,13^ Of the nearly 300 000 women diagnosed with breast cancer annually, about 83% have hormone receptor–positive tumors, which makes them eligible for adjuvant hormone therapy that is associated with increased survival and decreased risk of recurrence.^33^ Yet prior studies associated Medicaid insurance with lower use of both radiation and hormone therapy compared with other insurance.^12,13,18^ Using registry data alone, our findings confirmed these reported disparities. However, when using the linked Colorado APCD and CCCR, we did not observe disparities in radiation or hormone therapy. These findings were similar when we used longer follow-up times.

Our findings suggest that there are not radiation or hormone treatment disparities in Colorado between women with breast cancer covered by private and Medicaid insurance as has been previously reported by studies based on cancer registry data alone. This finding has serious implications for studies that use registry data alone and report disparities in treatment for Medicaid insured patients. Studies from Georgia that used medical records to assess treatment also reported fewer treatment disparities between Medicaid and privately insured women than studies using registry data alone.^23^ Statistically significant differences between urban and rural residents also disappeared when using the APCD data.

We hypothesize that treatment underreporting could stem from (1) clinicians who treat a higher share of patients insured by Medicaid may practice in health systems with insufficient support for registry reporting; (2) treatment plans may be less well documented for patients covered by Medicaid compared with privately insured patients; and/or (3) patients with Medicaid may take longer or may be less likely to complete treatment plans after surgery relative to privately insured patients, although raw frequencies did not support this hypothesis (Table 4). When treatment is delayed, it is less likely to be reported to the central registry. Underreporting might be mitigated through electronic data extraction from medical records or more support for the registry. The CCCR is working with facilities to ameliorate some of these issues. More support may also be needed for women with Medicaid to complete treatment in a timely manner.

**Table 4.** Frequencies of Having Radiation Therapy or Hormone Therapy Following Last Surgery by Months Depending on Information Source, CCCR-APCD, 2012 to 2017

Months following last surgery	Medicaid (n=1408)		Private (n=1984)	
	No. (%)	Cumulative %	No. (%)	Cumulative %
**Eligible for radiation therapy** ^a^ ** (n=3392)**				
0	79 (5.61)	5.61	156 (7.86)	7.86
1	218 (15.48)	21.09	370 (18.65)	26.51
2	99 (7.03)	28.13	123 (6.20)	32.71
3	34 (2.41)	30.54	53 (2.67)	35.38
4	83 (5.89)	36.43	114 (5.75)	41.13
5	103 (7.32)	43.75	137 (6.91)	48.03
6	128 (9.09)	52.84	167 (8.42)	56.45
7	89 (6.32)	59.16	102 (5.14)	61.59
8	83 (5.89)	65.06	54 (2.72)	64.31
9	31 (2.2)	67.26	29 (1.46)	65.78
10 (End-of-study censoring)	461 (32.74)	100	679 (34.22)	100
**Eligible for hormone therapy** ^a^ ** (n=2823)**				
0	499 (43.17)	43.17	649 (38.93)	38.93
1	310 (26.82)	69.98	511 (30.65)	69.59
2	130 (11.25)	81.23	211 (12.66)	82.24
3	70 (6.06)	87.28	132 (7.92)	90.16
4	33 (2.85)	90.14	46 (2.76)	92.92
5	23 (1.99)	92.13	12 (0.72)	93.64
6-7^b^	21 (1.82)	93.94	18 (1.08)	94.72
8-9^b^	11 (0.96)	94.90	15 (0.90)	95.62
10 (End-of-study censoring)	59 (5.10)	100	73 (4.38)	100

Abbreviations: APCD, All Payer Claims Database; CCCR, Colorado Central Cancer Registry.

^a^ Footnotes to Table 1 apply.

^b^ We collapsed 2 months in 1 category to achieve a displayed cell value that is greater than 10.

Known limitations in cancer registries make assessments of treatment disparities difficult.^34^ Initiatives such as the SEER-Medicaid linkage will aid in a more accurate assessment of Medicaid,^35^ but a comprehensive data infrastructure is needed to fully assess health outcomes across insurance types. We eliminated the possibility of insurance misclassification, a common problem with cancer registries, by linking claims data to the registry and identifying insurance type in the APCD data.^34^ In contrast, researchers using registry data alone may wrongly attribute observed disparities to a particular insurance type or being uninsured.

Medicaid is the primary insurer for qualifying low-income individuals who do not have other forms of health insurance. Medicaid is also the primary insurer for women who have breast cancer detected under the NBCCEDP. As more states evaluate Medicaid expansions, it is critical that policy makers accurately understand Medicaid’s value. Medicaid often enrolls patients following a cancer diagnosis at a later-stage disease,^36^ which can bias assessments of Medicaid treatment toward poor outcomes when untimely diagnosis is an important contributing factor.^37^ These data can only be found in cancer registries and medical records that are an important complement to claims data. In addition, patients enrolled in Medicaid have more comorbid conditions relative to privately insured patients.^38^ Despite these challenges, our findings suggest that patients enrolled in Medicaid seem to receive treatment at a rate similar to privately insured patients when registry data are supplemented with claims. This assessment suggests that disparities attributable to Medicaid may be overstated in some studies, and that Medicaid’s value to patients with breast cancer is higher than previously thought.

### Limitations

The main limitation of this study is that states vary in cancer registry quality, despite NAACCR certification and standards, and in the management of Medicaid. Therefore, these findings may not generalize to other states or to other types of cancer and treatment. Similar research should be conducted for other cancers and treatments and in other states with different approaches to Medicaid such as states without Medicaid expansion or higher rates of Medicaid managed care. Colorado is one of the few states that does not enroll most patients with Medicaid in commercial managed care plans. The CIVHC reports that 100% of Medicaid claims are captured in the APCD,^39^ and our assessments of the linked data corroborate this report.^27^ Thus, Medicaid managed care is not likely to affect completeness of claims in Colorado.^20^ In addition, our sample selection criteria omitted women who did not receive surgery, biasing the sample toward a treated group. However, only a small and statistically insignificant difference in surgical treatment was observed.

Next, we did not measure whether treatment was completed. Prior studies reported that women covered by Medicaid experience interruptions during therapy and are less likely to complete a minimum course of treatment.^40^ Last, the APCD does not include all privately insured women. Private payers that are covered by ERISA, approximately one-third of private payers in Colorado, are not required to report claims to the APCD, although some voluntarily do so. In addition, private payers covering fewer than 100 enrolled individuals are also not required to submit claims. Therefore, we cannot rule out that the inclusion of these women would change the findings. However, it is unlikely that these women are systematically different from other privately insured women in the APCD.

## Conclusions

In this cohort study of women newly diagnosed with breast cancer and covered by either Medicaid or private insurance in Colorado, there was no evidence of treatment disparities for radiation or hormone therapy between the 2 groups. Findings of this study demonstrated the utility of linked APCD and cancer registry data and suggest that prior studies of treatment disparities between Medicaid and privately insured women that relied solely on registry data may be overstated. The APCDs offer an opportunity to expand the existing health data infrastructure to address patient outcomes, policy, and health care delivery research questions. This study is 1 such example of how APCDs, when merged with a population-based registry, can be used to challenge prior findings based on more limited data. Our approach is a prototype for how other states can use the data to address similar questions. This study also has implications for cancer surveillance. Additional support may be needed to improve cancer reporting among clinicians who treat a higher proportion of patients covered by Medicaid and those who provide care in rural settings.

## Notes

Supplement 1. eFigure 1. Sample derivation–Radiation therapy analysis sample eFigure 2. Sample derivation–Hormonal therapy analysis sample eAppendix of Codes eTable 1. Likelihood of radiation therapy within 12 months following last surgery depending on information source, CCCR-APCD, 2012-2017, N=3219 eTable 2. Likelihood of hormonal therapy within 12 months following last surgery depending on information source, CCCR-APCD, 2012-2017, N=2690 eTable 3. Likelihood of radiation therapy within 9 months following last surgery depending on information source, CCCR-APCD, 2012-2017, N=3138 eTable 4. Likelihood of hormonal therapy within 9 months following last surgery depending on information source, CCCR-APCD, 2012-2017, N=2619

Supplement 2. Data Sharing Statement

## References

[1] Finkelstein A, Taubman S, Wright B, ; Oregon Health Study Group. The Oregon Health Insurance Experiment: evidence from the first year. Q J Econ. 2012;127(3):1057-1106. doi:10.1093/qje/qjs020 23293397 PMC3535298

[2] Card D, Dobkin C, Maestas N. The impact of nearly universal insurance coverage on health care utilization: evidence from Medicare. Am Econ Rev. 2008;98(5):2242-2258. doi:10.1257/aer.98.5.2242 19079738 PMC2600774

[3] Walker GV, Grant SR, Guadagnolo BA, . Disparities in stage at diagnosis, treatment, and survival in nonelderly adult patients with cancer according to insurance status. J Clin Oncol. 2014;32(28):3118-3125. doi:10.1200/JCO.2014.55.6258 25092774 PMC4876335

[4] Hsu CD, Wang X, Habif DV Jr, Ma CX, Johnson KJ. Breast cancer stage variation and survival in association with insurance status and sociodemographic factors in US women 18 to 64 years old. Cancer. 2017;123(16):3125-3131. doi:10.1002/cncr.30722 28440864

[5] Niu X, Roche LM, Pawlish KS, Henry KA. Cancer survival disparities by health insurance status. Cancer Med. 2013;2(3):403-411. doi:10.1002/cam4.84 23930216 PMC3699851

[6] Xie E, Colditz GA, Lian M, . Timing of Medicaid enrollment, late-stage breast cancer diagnosis, treatment delays, and mortality. J Natl Cancer Inst Cancer Spectr. 2022;6(3):pkac031. doi:10.1093/jncics/pkac031 35583139 PMC9113434

[7] Ayanian JZ, Kohler BA, Abe T, Epstein AM. The relation between health insurance coverage and clinical outcomes among women with breast cancer. N Engl J Med. 1993;329(5):326-331. doi:10.1056/NEJM199307293290507 8321261

[8] Berrian JL, Liu Y, Lian M, Schmaltz CL, Colditz GA. Relationship between insurance status and outcomes for patients with breast cancer in Missouri. Cancer. 2021;127(6):931-937. doi:10.1002/cncr.33330 33201532 PMC9386891

[9] Dawes AJ, Louie R, Nguyen DK, . The impact of continuous Medicaid enrollment on diagnosis, treatment, and survival in six surgical cancers. Health Serv Res. 2014;49(6):1787-1811. doi:10.1111/1475-6773.12237 25256223 PMC4254125

[10] Tsui J, DeLia D, Stroup AM, . Association of Medicaid enrollee characteristics and primary care utilization with cancer outcomes for the period spanning Medicaid expansion in New Jersey. Cancer. 2019;125(8):1330-1340. doi:10.1002/cncr.31824 30561793

[11] Adams EK, Chien LN, Gabram-Mendola SG. Treatment patterns among medicaid-eligible women with breast cancer in georgia: are patterns different under the breast and cervical cancer prevention and treatment act? J Oncol Pract. 2012;8(1):46-52. doi:10.1200/JOP.2011.000221 22548011 PMC3266316

[12] Freedman RA, Virgo KS, He Y, . The association of race/ethnicity, insurance status, and socioeconomic factors with breast cancer care. Cancer. 2011;117(1):180-189. doi:10.1002/cncr.25542 20939011

[13] Foley KL, Kimmick G, Camacho F, Levine EA, Balkrishnan R, Anderson R. Survival disadvantage among Medicaid-insured breast cancer patients treated with breast conserving surgery without radiation therapy. Breast Cancer Res Treat. 2007;101(2):207-214. doi:10.1007/s10549-006-9280-2 16838114

[14] Medicaid.gov. June 2022 Medicaid & CHIP Enrollment Data Highlights. Accessed October 21, 2022. https://www.medicaid.gov/medicaid/program-information/medicaid-and-chip-enrollment-data/report-highlights/index.html

[15] Kaiser Family Foundation. Health Coverage by Race and Ethnicity, 2010-2019. Accessed August 3, 2022. https://www.kff.org/racial-equity-and-health-policy/issue-brief/health-coverage-by-race-and-ethnicity/

[16] Hoppe EJ, Hussain LR, Grannan KJ, Dunki-Jacobs EM, Lee DY, Wexelman BA. Racial disparities in breast cancer persist despite early detection: analysis of treatment of stage 1 breast cancer and effect of insurance status on disparities. Breast Cancer Res Treat. 2019;173(3):597-602. doi:10.1007/s10549-018-5036-z 30390216

[17] Bradley CJ, Sabik LM, Entwistle J, Stevens JL, Enewold L, Warren JL. Role of Medicaid in early detection of screening-amenable cancers. Cancer Epidemiol Biomarkers Prev. 2022;31(6):1202-1208. doi:10.1158/1055-9965.EPI-21-1077 35322273 PMC9167742

[18] Hassett MJ, Schymura MJ, Chen K, Boscoe FP, Gesten FC, Schrag D. Variation in breast cancer care quality in New York and California based on race/ethnicity and Medicaid enrollment. Cancer. 2016;122(3):420-431. doi:10.1002/cncr.29777 26536043 PMC4724235

[19] Jagsi R, Abrahamse P, Hawley ST, Graff JJ, Hamilton AS, Katz SJ. Underascertainment of radiotherapy receipt in Surveillance, Epidemiology, and End Results registry data. Cancer. 2012;118(2):333-341. doi:10.1002/cncr.26295 21717446 PMC3224683

[20] Bradley CJ, Liang R, Jasem J, Lindrooth RC, Sabik LM, Perraillon MC. Cancer treatment data in central cancer registries: when are supplemental data needed? Cancer Inform. 2022;21:11769351221112457. doi:10.1177/11769351221112457 35923286 PMC9340909

[21] Grant SR, Walker GV, Koshy M, . Impact of insurance status on radiation treatment modality selection among potential candidates for prostate, breast, or gynecologic brachytherapy. Int J Radiat Oncol Biol Phys. 2015;93(5):968-975. doi:10.1016/j.ijrobp.2015.08.036 26452570

[22] Churilla TM, Egleston B, Bleicher R, Dong Y, Meyer J, Anderson P. Disparities in the local management of breast cancer in the US according to health insurance status. Breast J. 2017;23(2):169-176. doi:10.1111/tbj.12705 27797159

[23] Guy GP Jr, Lipscomb J, Gillespie TW, Goodman M, Richardson LC, Ward KC. Variations in guideline-concordant breast cancer adjuvant therapy in rural Georgia. Health Serv Res. 2015;50(4):1088-1108. doi:10.1111/1475-6773.12269 25491350 PMC4545348

[24] Center for Health and Environmental Data. Colorado Central Cancer Registry. Accessed February 13, 2023. https://cdphe.colorado.gov/center-for-health-and-environmental-data/registries-and-vital-statistics/colorado-central-cancer

[25] Center for Improving Value in Health Care. CO APCD Overview. Accessed February 13, 2023. https://www.civhc.org/get-data/co-apcd-info/

[26] Center for Improving Value in Health Care. CO APCD Insights Dashboard. Accessed February 13, 2023. https://www.civhc.org/get-data/whats-in-the-co-apcd/

[27] Perraillon MC, Liang R, Sabik LM, Lindrooth RC, Bradley CJ. The role of all-payer claims databases to expand central cancer registries: experience from Colorado. Health Serv Res. 2022;57(3):703-711. doi:10.1111/1475-6773.13901 34743320 PMC9108037

[28] National Comprehensive Cancer Network. NCCN Guidelines Breast Cancer. Accessed February 13, 2023. https://www.nccn.org/guidelines/guidelines-detail?category=1&id=1419

[29] Equator Network. STROBE Checklist Cohort. Accessed February 13, 2023. https://www.equator-network.org/wp-content/uploads/2015/10/STROBE_checklist_v4_cohort.pdf

[30] National Cancer Institute Surveillance, Epidemiology, and End Results Program. SEER*Rx - Interactive Antineoplastic Drugs Database. Updated September 30, 2014. Accessed January 20, 2022. https://seer.cancer.gov/tools/seerrx/

[31] U.S. Department of Agriculture. 2010 Rural-Urban Commuting Area (RUCA) Codes. Accessed February, 2022. https://www.ers.usda.gov/data-products/rural-urban-commuting-area-codes/documentation/

[32] Norton EC, Dowd BE, Maciejewski ML. Marginal effects-quantifying the effect of changes in risk factors in logistic regression models. JAMA. 2019;321(13):1304-1305. doi:10.1001/jama.2019.1954 30848814

[33] Fu F, Yu L, Zeng B, . Association of adjuvant hormone therapy timing with overall survival among patients with hormone receptor-positive human epidermal growth factor receptor-2-negative early breast cancer without chemotherapy. JAMA Netw Open. 2022;5(2):e2145934. doi:10.1001/jamanetworkopen.2021.45934 35166783 PMC8848199

[34] Sabik LM, Bradley CJ. Understanding the limitations of cancer registry insurance data-implications for policy. JAMA Oncol. 2018;4(10):1432-1433. doi:10.1001/jamaoncol.2018.2436 30054631

[35] Warren JL, Benner S, Stevens J, . Development and evaluation of a process to link cancer patients in the SEER Registries to National Medicaid Enrollment Data. J Natl Cancer Inst Monogr. 2020;2020(55):89-95. doi:10.1093/jncimonographs/lgz035 32412075 PMC7868030

[36] Bradley CJ, Stevens JL, Enewold L, Warren JL. Stage and mortality of low-income patients with cancer: evidence from SEER-Medicaid. Cancer. 2021;127(2):229-238. doi:10.1002/cncr.33207 33107990

[37] Ellis L, Canchola AJ, Spiegel D, Ladabaum U, Haile R, Gomez SL. Trends in cancer survival by health insurance status in California from 1997 to 2014. JAMA Oncol. 2018;4(3):317-323. doi:10.1001/jamaoncol.2017.3846 29192307 PMC5885831

[38] Chapel JM, Ritchey MD, Zhang D, Wang G. Prevalence and medical costs of chronic diseases among adult Medicaid beneficiaries. Am J Prev Med. 2017;53(6S2)(suppl 2):S143-S154. doi:10.1016/j.amepre.2017.07.019 29153115 PMC5798200

[39] Center for Improving Value in Health Care. Medicaid Data in the CO APCD. Accessed February 13, 2023. https://www.civhc.org/2022/10/12/medicaid-data-in-the-co-apcd/

[40] Ramsey SD, Zeliadt SB, Richardson LC, . Discontinuation of radiation treatment among Medicaid-enrolled women with local and regional stage breast cancer. Breast J. 2010;16(1):20-27. doi:10.1111/j.1524-4741.2009.00865.x 19929888

