# Supplementary material for: Treatment Disparities in Radiation and Hormone Therapy Among Women Covered by Medicaid vs Private Insurance in Cancer Registry and Claims Data
Source: JAMA Health Forum. 2023 May 5;4(5):e230673. doi: 10.1001/jamahealthforum.2023.0673 (PMC10163382; doi:10.1001/jamahealthforum.2023.0673)
Supplement: Supplement 1. — eFigure 1. Sample derivation–Radiation therapy analysis sample eFigure 2. Sample derivation–Hormonal therapy analysis sample eAppendix of Codes eTable 1. Likelihood of radiation therapy within 12 months following last surgery depending on information source, CCCR-APCD, 2012-2017, N=3219 eTable 2. Likelihood of hormonal therapy within 12 months following last surgery depending on information source, CCCR-APCD, 2012-2017, N=2690 eTable 3. Likelihood of radiation therapy within 9 months following last surgery depending on information source, CCCR-APCD, 2012-2017, N=3138 eTable 4. Likelihood of hormonal therapy within 9 months following last surgery depending on information source, CCCR-APCD, 2012-2017, N=2619 [file jamahealthforum-e230673-s001.pdf]

Supplemental Online Content

Bradley CJ, Sabik LM, Liang R, Lindrooth RC, Perraillon MC. Treatment disparities in radiation and hormone therapy among women covered by Medicaid vs private insurance in cancer registry and claims data. *JAMA Health Forum*. 2023;4(5):e230673. doi:10.1001/jamahealthforum.2023.0673

eFigure 1. Sample derivation–Radiation therapy analysis sample

eFigure 2. Sample derivation–Hormonal therapy analysis sample

eAppendix of Codes

eTable 1. Likelihood of radiation therapy within 12 months following last surgery depending on information source, CCCR-APCD, 2012-2017, N=3219

eTable 2. Likelihood of hormonal therapy within 12 months following last surgery depending on information source, CCCR-APCD, 2012-2017, N=2690

eTable 3. Likelihood of radiation therapy within 9 months following last surgery depending on information source, CCCR-APCD, 2012-2017, N=3138

eTable 4. Likelihood of hormonal therapy within 9 months following last surgery depending on information source, CCCR-APCD, 2012-2017, N=2619

This supplemental material has been provided by the authors to give readers additional information about their work.

© 2023 Bradley CJ, et al. *JAMA Health Forum.*

eFigure 1. Sample derivation – Radiation therapy analysis sample

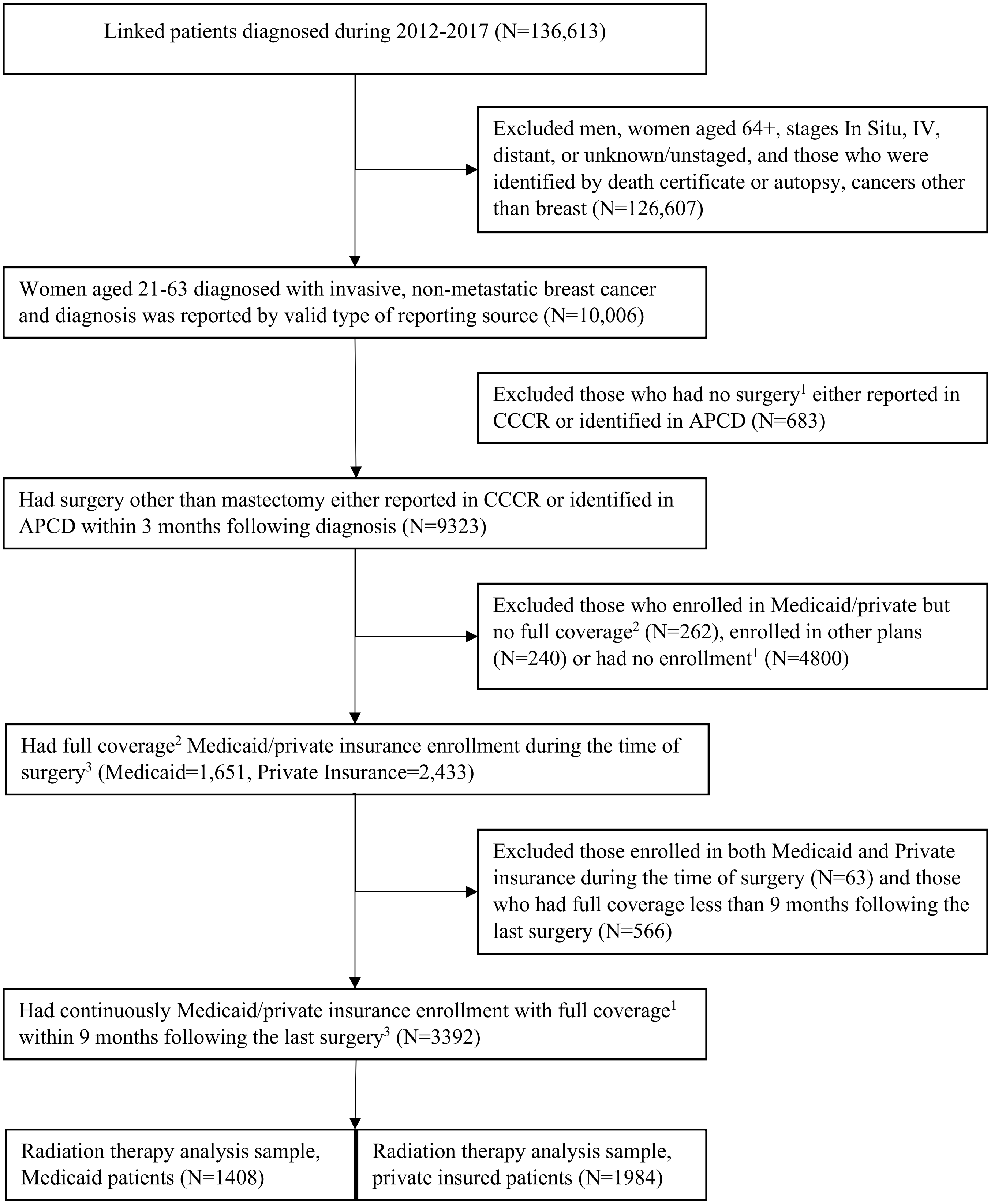

© 2023 Bradley CJ, et al. *JAMA Health Forum.*

Notes: CCCR= Colorado Central Cancer Registry; APCD=All Payer Claims Database
[1] Surgery for radiation therapy analysis was defined as biopsy (except fine needle biopsies), breast conserving surgery, lumpectomy, lymphadenectomy and sentinel lymphadenectomy.
[2] Full coverage defined as enrolled in plans with medical and pharmacy coverage.
[3] Time of surgery defined as the month of last surgery identified from APCD claims. If APCD claims were not available, we applied the surgery date from CCCR. The month of last surgery from APCD was based on the service date of the last surgery within 3 months of the first surgery, which was identified from any time after diagnosis in the APCD claims data.

© 2023 Bradley CJ, et al. *JAMA Health Forum.*

eFigure 2. Sample derivation – Hormonal therapy analysis sample

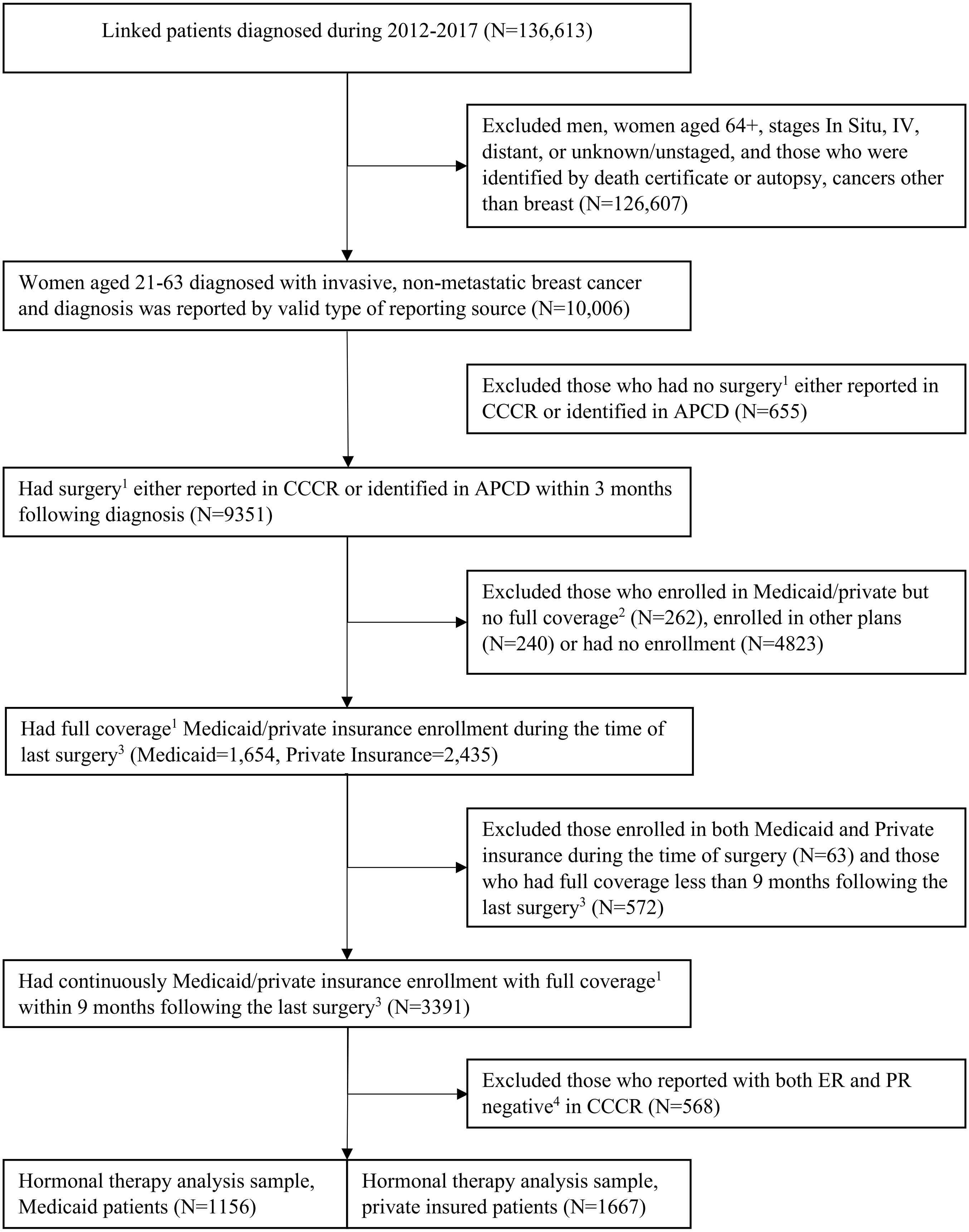

© 2023 Bradley CJ, et al. *JAMA Health Forum.*

Notes: CCCR= Colorado Central Cancer Registry; APCD=All Payer Claims Database; ER=estrogen receptor; PR=progesterone receptor.
[1] Surgery for hormonal therapy analysis, was defined as biopsy (except fine needle biopsy), mastectomy, breast conserving surgery, lumpectomy, lymphadenectomy and sentinel lymphadenectomy.
[2]Full coverage defined as enrolled in plans with medical and pharmacy benefit coverage.
[3] Time of surgery defined as the month of last surgery identified from APCD claims. If APCD data was not available, we applied the surgery date from CCCR. The month of last surgery from APCD was based on the service date of the last surgery within 3 months of the first surgery, identified from any time after diagnosis in the APCD claims data.
[4] Patients included in hormonal therapy analysis sample required women who were hormone receptor-positive with either ER or PR positive.

© 2023 Bradley CJ, et al. *JAMA Health Forum.*

eAppendix of Codes

Radiation therapy

CPT/HCPCS

19296,19297,19298,20555,20660,31463,32553,41019,49411,49412,52250,55859,55860,55875,55876,55920,57155,57156,58346,61720,61735,61770,61781,61782,61783,61793,61795,61796,61797,61798,617
99,61800,63620,63621,73670,76950,76965,77370,77370,77371,77372,77373,77380,77381,77385,77386,77387,77399,77400,77401,77402,77403,77404,77405,77406,77407,77408,77409,77410,77411,77412,
77413,77414,77415,77416,77417,77418,77421,77422,77423,77470,77520,77522,77523,77525,77750,77761,77762,77763,77776,77777,77778,77781,77782,77783,77784,77785,77786,77787,77789,77790,777
99,79005,79030,79035,79100,79101,79200,79300,79400,79403,79420,79440,79445,79999,0073T,0082T,0182T,0190T,0197T,77295,77300,77301,77332,77333,77334,77338,79900,0083T,C1350,C2632,C97
14,C9715,C9726,C9728,G0173,G0174,G0178,G0243,G0251,G0256,G0273,G0274,G0339,G0339,G0340,G0340,G0458,G6003,G6004,G6005,G6006,G6007,G6008,G6009,G6010,G6011,G6012,G6013,G6014
,G6015,G6016,S2270,S8049,A4650,A9606,A9699,C1325,C1348,C1700,C1701,C1702,C1703,C1704,C1705,C1706,C1707,C1708,C1709,C1710,C1711,C1712,C1715,C1716,C1717,C1718,C1719,C1720,C172
8,C1790 ,C1791,C1792,C1793,C1794,C1795,C1796,C1797,C1798,C1799,C1800,C1801,C1802,C1803,C1804,C1805,C1806,C2616,C2633,C2634,C2635,C2636,C2637,C2638,C2639,C2640,C2641,C2642,C2
643,C2644,C2645,C2698,C2699,Q3001

ICD-9/10 Diagnosis / Procedure codes

V58.0,V66.1,V67.1,Z51.0,Z.08,92.2,92.21,92.22,92.23,92.24,92.25,92.26,92.27,92.28,92.29,92.41,D0xxxxx,D7xxxxx,D8xxxxx,D9xxxxx,DBxxxxx,DDxxxxx,DFxxxxx,DGxxxxx,DHxxxxx,DMxxxxx

Revenue codes

330,333,334

MS/APR-DRG codes

849,692

Brest cancer surgery

Breast-conserving surgery

CPT/HCPCS

19120,19125,19126,19160,19301,19162,19302

ICD-9/10 Procedure codes

85.20,85.21,85.22,85.23,0HBT0ZZ,0HBT3ZZ,0HBT7ZZ,0HBT8ZZ,0HBTXZZ,0HBU0ZZ,0HBU3ZZ,0HBU7ZZ,0HBU8ZZ,0HBUXZZ,0HBV0ZZ,0HBV3ZZ,0HBV7ZZ,0HBV8ZZ,0HBVXZZ,0HBW0ZZ,
0HBW3ZZ,0HBW7ZZ,0HBW8ZZ,0HBWXZZ,0HBX0ZZ,0HBX3ZZ,0HBX7ZZ,0HBX8ZZ,0HBXXZZ,0HBY0ZZ,0HBY3ZZ,0HBY7ZZ,0HBY8ZZ,0HBYXZZ,0H5T0ZZ,0H5T3ZZ,0H5T7ZZ,0H5T8ZZ,0
H5TXZZ,0H5U0ZZ,0H5U3ZZ,0H5U7ZZ,0H5U8ZZ,0H5UXZZ,0H5V0ZZ,0H5V3ZZ,0H5V7ZZ,0H5V8ZZ,0H5VXZZ,0H5W0ZZ,0H5W3ZZ,0H5W7ZZ,0H5W8ZZ,0H5WXZZ,0H5X0ZZ,0H5X3ZZ,0H5X7
ZZ,0H5X8ZZ,0H5XXZZ

Mastectomy

CPT/HCPCS

19180,19182,19303,19304,19200,19220,19240,19305,19306,19307

ICD-9/10 Procedure codes

85.33,85.34,85.35,85.36,85.40,85.41,85.42,85.43,85.44,85.45,85.46,85.47,85.48,0HDT0ZZ,0HDU0ZZ,0HDV0ZZ,0HDY0ZZ,0HTT0ZZ,0HTU0ZZ,0HTV0ZZ,0HTY0ZZ,0HTWXZZ,0HTXX

MS/APR-DRG codes

582,583

Axillary node dissection/lymph node resection/lymphadenectomy/Sentinel lymphadenectomy

CPT/HCPCS

38790,38792,38900,78195,38500,38510,38520,38525,38530,38542,38740,38745

© 2023 Bradley CJ, et al. *JAMA Health Forum.*

ICD-9/10 Procedure codes

40.22,40.23,40.29,40.3,40.50,40.51,07T50ZZ,07T60ZZ,07T70ZZ,07T80ZZ,07T90ZZ,0KTH0ZZ,0KTJ0ZZ

Biopsy (except fine needle biopsy)

CPT/HCPCS

19000,19001,19081,19082,19083,19084,19085,19086,19100,19101,19102,19103

ICD-9/10 Procedure codes

85.12,0H9T00Z,0H9T30Z,0H9T70Z,0H9T80Z,0H9U00Z,0H9U30Z,0H9U70Z,0H9U80Z,0H9V00Z,0H9V30Z,0H9V70Z,0H9V80Z,0H9W00Z,0H9W30Z,0H9W70Z,0H9W80Z,0H9WX0Z,0H9X00Z,0H9X3
0Z,0H9X70Z,0H9X80Z,0H9XX0Z,0H9T0ZZ,0H9T3ZZ,0H9T7ZZ,0H9T8ZZ,0H9TZZZ,0H9U0ZZ,0H9U3ZZ,0H9U7ZZ,0H9U8ZZ,0H9UZZZ,0H9V0ZZ,0H9V3ZZ,0H9V7ZZ,0H9V8ZZ,0H9W0ZZ,0H9W3
ZZ,0H9W7ZZ,0H9W8ZZ,0H9WZZZ,0H9X0ZZ,0H9X3ZZ,0H9X7ZZ,0H9X8ZZ,0H9XZZZ,0HBT0ZX,0HBT3ZX,0HBT7ZX,0HBT8ZX,0HBTXZX,0HBU0ZX,0HBU3ZX,0HBU7ZX,0HBU8ZX,0HBUXZ
X,0HBV0ZX,0HBV3ZX,0HBV7ZX,0HBV8ZX,0HBVXZX,0HBW0ZX,0HBW3ZX,0HBW7ZX,0HBW8ZX,0HBWXZX,0HBX0ZX,0HBX3ZX,0HBX7ZX,0HBX8ZX,0HBXXZX,0HBY0ZX,0HBY3ZX,0H
BY7ZX,0HBY8ZX,0HBYXZX

MS/APR-DRG codes

584,585

Hormonal therapy

CPT/HCPCS

C9216,C9237,C9430,C9454,J0128,J1020,J1030,J1040,J1050,J1051,J1094,J1100,J1380,J1675,J1710,J1720,J1725,J1726,J1729,J1930,J1950,J2353,J2354,J2502,J2650,J2920,J2930,J3120,J3121,J3130,J3240,J3
300,J3301,J3315,J7506,J7509,J7510,J7512,J7684,J8540,J9155,J9202,J9217,J9218,J9219,J9225,J9226,J9395,S0156,S0165,S0170,S0179,S0187,S0190,J1620,J2675,J0800,J2725,J2940,J8515,J9171,G0356

ICD-9/10 Procedure codes

99.24,3E013VJ,3E033VJ,3E043VJ,3E053VJ,3E063VJ

NDC codes

63739-0161-10,68788-7102-09,68788-7102-01,68788-7102-06,68788-7102-03,70518-0920-00,70720-0951-30,42195-0121-06,42195-0149-12,58463-0010-08,58463-0014-01,58463-0015-01,58463-0016-
01,58463-0017-01,67296-0326-01,57894-0155-12,57894-0199-06,60760-0179-35,60760-0255-60,60760-0255-21,60760-0255-40,70518-0948-00,70121-1168-01,70121-1169-01,70720-0950-36,71205-0012-
21,70518-1088-00,47335-0401-81,69097-0316-02,71205-0013-49,70518-1116-00,70518-1120-00,70934-0101-21,70518-1167-00,70518-1119-00,69101-0410-01,16714-0816-01,16714-0816-02,48102-0048-
01,68788-7142-01,68788-7142-03,68788-7142-05,48102-0045-01,48102-0046-01,70069-0021-25,68382-0916-01,68382-0916-05,68382-0916-34,68382-0917-01,68382-0917-05,68382-0917-11,68382-0917-
77,68382-0918-01,68382-0918-05,68382-0918-18,68382-0918-77,68382-0919-01,68382-0919-05,68382-0919-11,68382-0919-77,70771-1348-01,70771-1348-03,70771-1348-05,70771-1349-01,70771-1349-
04,70771-1349-05,70771-1349-08,70771-1350-01,70771-1350-04,70771-1350-05,70771-1350-07,70771-1351-01,70771-1351-04,70771-1351-05,70771-1351-08,43975-0315-10,50268-0694-15,70069-0024-
25,70069-0023-25,70069-0022-25,62559-0680-30,47335-0485-83,47335-0485-18,47335-0485-08,00009-0698-02,00002-4165-02,00002-4165-07,00002-4165-30,00002-4165-34,00002-4165-61,00002-4165-
79,00002-4165-99,00003-0315-05,00003-0315-20,00009-0039-06,00009-0039-28,00009-0039-30,00009-0039-32,00009-0039-33,00143-9289-01,00143-9290-01,00143-9291-01,00310-7720-10,00378-0642-
01,00378-0642-05,00378-0642-10,00378-6921-91,00469-0625-99,00469-0725-60,00517-0420-01,00517-0440-01,00527-2935-37,00591-5019-02,00615-1542-39,00703-0241-01,00703-0243-01,00703-0245-
01,00781-3079-12,00781-3492-12,00904-6914-61,00904-6948-04,15054-1060-03,15054-1060-04,15054-1090-03,15054-1090-04,15054-1120-03,15054-1120-04,16714-0118-02,16714-0130-01,16714-0130-
25,16714-0140-01,16714-0150-01,16714-0963-01,16729-0436-30,17856-0032-01,17856-0759-01,17856-0759-02,17856-0759-03,17856-0759-04,17856-0759-05,23155-0685-31,23155-0686-31,23155-0687-
41,23155-0688-41,23155-0689-41,23594-0505-01,23594-0505-02,23594-0505-21,23594-0505-48,23594-0505-50,25021-0462-74,33261-0667-30,33261-0667-60,51662-1483-01,00121-0902-04,42291-0085-
30,42291-0085-90,42806-0400-01,42806-0400-21,44278-0025-30,43063-0383-06,43063-0383-15,69238-1754-06,42291-0073-60,50268-0075-15,60687-0455-21,43598-0358-04,72606-0566-01,69539-0049-
92,68001-0489-07,72205-0030-92,50090-2453-00,50090-2453-01,51991-0620-10,51991-0620-33,63629-5269-01,63629-5269-02,63629-5269-03,63629-5269-04,63629-5269-05,63629-5269-06,63629-5269-
07,63629-5269-08,63629-5269-09,70934-0488-10,59651-0236-30,59651-0236-90,70518-2420-00,62559-0890-30,70518-2993-00,49999-0059-06,49999-0059-12,49999-0059-30,49999-0059-60,49999-0059-
90,50090-2313-02,50090-2313-03,61786-0979-01,61786-0979-52,70518-0532-00,70518-0532-01,55150-0304-25,72572-0122-25,55150-0305-10,71872-7239-01,63629-4788-01,63629-4788-02,63629-4788-
03,63629-4788-04,63629-4788-05,50090-1704-00,50090-1704-01,50090-1704-02,70934-0541-30,70934-0541-90,50090-1882-00,50090-1882-01,50090-1876-00,50090-1876-02,50090-0627-01,50090-0627-
02,50090-0627-03,70518-3019-00,70121-1452-01,70121-1452-05,71921-0190-33,51991-0005-33,51991-0005-90,63629-2056-01,71335-1424-01,71335-1424-02,71335-1424-03,71335-1424-04,71335-1424-
05,71335-1424-06,70771-1185-00,70771-1185-01,70771-1185-03,70771-1185-04,70771-1185-05,70771-1185-06,70771-1185-09,70771-1184-01,70771-1184-03,70771-1184-04,70771-1184-05,70771-1184-
06,70771-1184-08,70771-1184-09,63739-0269-10,63739-0269-42,68382-0826-01,68382-0826-05,68382-0826-06,68382-0826-14,68382-0826-16,68382-0826-28,68382-0826-77,51662-1297-01,51662-1297-
03,50268-0476-15,50419-0395-01,50419-0395-72,68842-0301-12,71731-6121-01,71288-0555-86,72603-0105-02,43598-0262-02,68001-0484-85,63323-0715-05,67457-0311-05,68462-0317-32,66993-0212-
38,72205-0050-30,69539-0152-30,00591-2433-15,00591-2434-15,00591-2451-15,00591-2501-15,00591-2435-15,00591-2436-15,70518-2994-00,70518-3046-00,71209-0082-01,71209-0082-05,71209-0082-
13,51662-1263-01,51662-1263-03,51662-1264-01,51662-1264-03,63629-4413-01,63629-4413-02,63629-4413-03,63629-4413-04,63629-4413-05,63629-4413-06,68382-0827-01,68382-0827-05,68382-0827-
06,68382-0827-10,68382-0827-14,68382-0827-16,68382-0827-77,59651-0299-60,70518-2721-00,59651-0300-30,59651-0300-90,42292-0057-03,68462-0135-08,48102-0047-01,48102-0047-20,42195-0270-

© 2023 Bradley CJ, et al. *JAMA Health Forum.*

07,71905-0400-11,79043-0200-25,70860-0211-74,68001-0424-85,70534-0002-01,59762-0055-01,59762-0055-02,59762-0058-01,59762-0058-02,59762-0058-04,72647-0331-01,72647-0331-04,70625-0237-
01,70625-0237-11,00002-4184-02,00002-4184-07,00002-4184-30,68180-0685-01,68788-7655-02,68180-0688-08,68180-0689-55,68180-0686-01,68180-0686-11,68180-0687-55,70518-2023-00,70518-2023-
01,62756-0452-36,62756-0452-37,60219-1707-01,60219-1707-03,60219-1707-05,60219-1708-01,60219-1708-05,42195-0221-06,42195-0721-21,42195-0490-12,55700-0854-21,51662-1370-01,61919-0827-
15,61919-0827-21,51662-1432-01,76420-0077-01,70069-0025-10,71872-7205-01,51662-1391-01,72893-0015-06,72893-0015-24,63187-0561-00,63187-0561-10,63187-0561-15,63187-0561-24,63187-0561-
30,63187-0561-60,63187-0561-90,63629-4127-01,63629-4127-02,63629-4127-03,63629-4127-04,63629-4127-05,63629-4127-06,63629-4127-07,63629-4127-08,63629-4129-01,63629-4129-02,63629-4129-
03,63629-4129-04,63629-4129-05,63629-4129-06,52959-0392-06,52959-0392-12,52959-0392-15,52959-0392-21,52959-0392-28,52959-0392-30,52959-0547-01,52959-0547-04,52959-0547-05,52959-0547-
07,52959-0547-10,52959-0547-11,52959-0547-12,52959-0547-16,52959-0547-18,52959-0547-20,52959-0547-21,52959-0547-28,52959-0547-30,52959-0547-50,50090-0088-00,50090-0088-04,70518-1534-
00,70518-1534-01,64239-0300-10,70518-2998-00,70518-3050-00,67296-1413-01,67296-1413-02,67296-1413-04,68047-0702-01,68047-0702-21,68047-0702-35,68047-0702-51,70518-2829-00,70934-0798-
30,54348-0701-30,72189-0198-90,54348-0702-90,61919-0198-30,61919-0198-60,63629-3755-01,63629-3755-02,63629-3755-03,63629-3755-04,63629-3755-05,63629-3755-06,70934-0215-30,70934-0215-
90,61919-0278-30,61919-0278-60,70934-0506-30,70934-0506-90,70934-0393-30,70518-2369-00,70518-2595-00,72189-0183-60,72189-0183-90,68788-7892-01,68788-7892-03,68788-7892-06,68788-7892-
09,50090-0167-00,50090-0167-05,60687-0511-01,64380-0971-06,64380-0970-25,00078-0909-61,57894-0184-12,00310-0201-14,00310-0201-30,00310-0201-37,00310-0201-97,00378-6034-05,00378-6034-
77,00378-6034-93,42292-0007-01,42292-0007-10,00093-0220-01,00093-0220-56,00310-0705-10,00310-0705-30,00310-0705-39,52125-0709-02,68382-0224-01,68382-0224-05,68382-0224-06,68382-0224-
10,68382-0224-30,68382-0224-77,49884-0373-01,60687-0289-01,60687-0289-11,00095-0086-01,00095-0086-10,00603-1147-56,00069-0177-01,00069-0177-02,00069-0178-01,00069-0178-02,00069-0179-
01,00069-0179-02,00069-0192-01,00069-0192-02,00069-4541-01,00069-4541-02,00069-4543-01,00069-4543-02,00069-4545-01,00069-4545-02,00069-4547-01,00069-4547-02,68071-3394-02,68071-3394-
03,68071-3394-06,68071-3394-09,00179-1999-01,00179-1999-30,00179-1999-60,00179-1999-70,00179-1999-88,00179-1999-90,00555-0886-01,00555-0886-02,00555-0886-04,00555-0886-14,00555-0887-
01,00555-0887-02,00555-0887-04,00555-0887-14,00046-1101-81,00046-1103-81,00591-2466-18,00310-0720-10,00310-0720-25,00310-0720-50,00310-0950-36,00310-0951-30,00009-0011-03,00009-0011-
04,00009-0047-04,00009-0047-22,00009-0047-25,00009-0047-26,00009-0047-27,00009-0049-02,00009-0056-02,00009-0056-03,00009-0056-04,00009-0056-05,00009-0064-04,00009-0064-06,00054-3722-
50,00054-3722-63,00143-1254-01,00143-1254-25,00603-3899-10,00603-3899-19,00603-3899-21,00603-3899-28,00603-3900-10,00603-3900-21,00603-3900-28,00603-3901-10,00603-3901-21,00378-6611-
85,00378-6611-93,00378-6612-85,00378-6612-93,00378-6614-85,00378-6614-88,00378-6614-93,00378-2071-05,00378-2071-93,00074-3346-03,00074-3473-03,00074-3642-03,00074-3683-03,00185-7400-
14,00185-7400-85,00555-0779-01,00555-0779-02,00555-0779-04,00555-0779-05,00555-0872-01,00555-0872-02,00555-0872-04,00555-0872-05,00555-0873-01,00555-0873-02,00555-0873-04,00555-0873-
05,68071-1870-01,00548-5401-25,00555-0606-02,00555-0606-03,00555-0606-04,00555-0607-02,00555-0607-03,00555-0607-04,00603-4593-15,00603-4593-21,00603-4593-28,00603-4593-32,00409-3217-
05,00409-3217-15,00088-1111-14,00078-0180-01,00078-0180-61,00078-0181-01,00078-0181-61,00078-0182-01,00078-0182-61,00078-0183-25,00078-0647-81,00178-0582-01,00178-0582-08,00603-1567-
56,00603-1567-58,00378-4710-22,00378-4715-22,00378-4730-22,68791-0104-04,68084-0075-11,68084-0075-21,00179-0224-01,00378-0144-05,00378-0144-91,70518-0306-00,70518-0306-01,70518-0307-
00,00591-5052-01,00591-5052-10,00591-5052-21,00591-5052-43,00591-5442-01,00591-5442-05,00591-5442-10,00591-5442-21,00591-5442-43,00591-5443-01,00591-5443-05,00591-5443-10,00603-5335-
10,00603-5335-21,00603-5335-28,00603-5335-32,00603-5336-10,00603-5336-21,00603-5336-28,00603-5336-32,00603-5337-15,00603-5337-21,00603-5337-28,00603-5337-31,00603-5337-32,00603-5339-
15,00603-5339-21,00603-5339-28,00603-5339-31,00603-5339-32,50090-2786-00,72974-0120-01,00054-0399-25,00527-2930-37,00527-2930-43,00527-2931-37,00527-2932-37,00527-2932-43,00527-2933-
37,00527-2933-41,00527-2934-37,00527-2934-41,57894-0150-12,57894-0150-25,57894-0195-06,57894-0195-15,62856-0601-22,00054-0164-13,00093-7536-56,00115-1261-01,00115-1261-03,00115-1261-
08,00115-1261-10,00179-0068-70,00781-5356-31,00904-6195-46,00904-6229-61,16571-0421-03,16729-0035-10,16729-0035-15,16729-0035-16,16729-0035-17,21695-0990-30,42043-0180-03,42254-0161-
30,42291-0105-30,50090-1193-00,50090-1918-00,50090-2005-00,50090-2118-00,51079-0323-01,51079-0323-06,51655-0638-53,54569-6198-00,54868-5000-00,54868-6130-00,54868-6130-01,55111-0647-
30,60258-0866-03,60429-0286-30,60429-0286-60,60429-0286-90,60505-2985-03,60687-0112-11,60687-0112-21,60763-0376-06,62033-0376-06,62175-0710-32,62756-0250-13,62756-0250-18,62756-0250-
83,62756-0250-88,63187-0080-30,63323-0129-30,63672-0015-00,65841-0743-06,65841-0743-10,66336-0533-30,66435-0415-30,67877-0171-10,67877-0171-30,68001-0155-04,68001-0155-08,68071-1682-
03,68382-0209-06,68382-0209-10,68788-6774-03,68788-6774-06,68788-6774-09,69189-0035-01,00187-5525-60,00187-5526-75,00378-6955-01,62856-0602-10,62856-0604-22,68682-0002-60,68682-0003-
10,00904-6019-46,16714-0571-01,16714-0571-02,16714-0571-03,16729-0023-01,16729-0023-10,41616-0485-08,41616-0485-18,41616-0485-83,41616-0485-88,42291-0168-30,42291-0168-50,47335-0485-
88,51079-0692-01,51079-0692-03,51991-0560-01,54868-4503-00,54868-6133-00,60429-0177-01,60429-0177-05,60429-0177-30,60429-0226-05,60429-0226-30,60505-2642-01,60505-2642-03,63629-5321-
01,63629-5321-02,63672-0005-00,65841-0613-01,65841-0613-05,65841-0613-06,65841-0613-10,65841-0613-30,67253-0191-03,67253-0191-10,68084-0374-11,68084-0374-21,69189-0298-01,00143-1202-
01,00143-9700-01,59115-0161-01,59115-0161-99,60429-0015-01,55566-8303-01,55566-8403-01,00054-3176-44,00054-3177-57,00054-3177-63,00054-4179-25,00054-4180-25,00054-4181-25,00054-4182-
25,00054-4182-31,00054-4183-25,00054-4184-25,00054-4186-25,00054-8174-25,00054-8175-25,00054-8176-25,00054-8179-25,00054-8180-25,00054-8181-25,00054-8183-25,00095-0087-35,00095-0088-
51,00095-0089-21,10544-0211-10,10544-0212-06,16590-0269-10,21695-0290-30,21695-0382-04,21695-0382-08,21695-0382-20,21695-0382-60,21695-0728-12,21695-0745-12,33261-0625-10,42195-0015-
01,42195-0105-06,42195-0105-12,42195-0150-21,42195-0150-49,43063-0266-07,49884-0084-01,49884-0085-01,49884-0086-01,49884-0087-01,49884-0129-01,50090-0087-00,50090-0087-03,50090-0089-
00,50090-0090-04,50090-0091-00,51655-0012-03,51655-0012-87,53217-0231-21,53217-0231-30,53217-0231-60,53217-0231-90,54569-0322-00,54569-0322-03,54569-0324-04,54569-0336-01,54569-5729-
00,54868-0218-00,54868-0218-01,54868-0218-02,54868-0218-03,54868-0218-04,54868-0218-05,54868-0218-06,54868-0218-07,54868-0218-08,54868-0218-09,54868-0916-00,54868-0927-00,54868-1744-
00,54868-3157-00,54868-3157-01,54868-5334-00,54868-5334-01,54868-5903-00,54879-0003-08,55154-3275-04,55154-3275-05,55154-3275-06,00009-0190-09,00009-0909-08,00009-0909-16,00009-0909-
20,00143-1410-10,00143-1410-25,00143-1473-01,00143-1473-05,00143-1473-10,00143-1473-25,00143-1475-01,00143-1475-10,00143-1475-25,00143-1477-01,00143-1477-05,00143-1477-10,00143-1477-
25,00172-4960-58,00172-4960-60,00172-4960-70,00378-0274-01,00378-0274-25,00378-0274-93,00378-7017-01,00378-7017-05,00378-7017-93,00409-5684-01,00409-5684-23,00409-5685-02,00409-5685-
23,00603-9388-56,00603-9388-58,00703-4014-11,00703-4014-18,00781-3029-75,00781-3030-75,00781-3031-75,00781-5409-01,00781-5409-10,00781-5409-31,00781-5409-64,24236-0217-13,24236-0217-
55,24236-0217-58,24236-0248-03,24236-0248-10,24236-0248-13,24236-0248-53,24236-0248-58,24236-0418-03,24236-0418-13,24236-0418-53,24236-0418-55,24236-0418-58,24236-0625-04,24236-0625-
13,24236-0625-20,42747-0327-07,42747-0327-30,42747-0327-72,43063-0109-10,43063-0415-01,43063-0415-30,43063-0415-36,43063-0415-42,43063-0415-46,43063-0415-55,43063-0415-60,43063-0415-
72,43063-0432-10,43063-0432-12,43063-0432-14,43063-0432-15,43063-0432-20,43063-0432-21,43063-0432-30,45802-0303-21,45802-0303-67,45802-0733-21,45802-0733-67,49349-0287-02,49349-0287-
08,49349-0550-02,49349-0550-03,49349-0550-08,49349-0550-13,49349-0550-50,49349-0607-02,49349-0607-03,49349-0607-08,49349-0607-20,49349-0607-57,49349-0717-02,49349-0725-02,49349-0755-
01,49349-0755-08,49349-0783-02,49349-0783-53,49349-0841-01,49349-0841-41,49349-0841-52,49349-0856-02,49349-0948-01,49349-0997-02,49999-0008-00,49999-0008-20,49999-0008-21,49999-0008-
30,49999-0008-40,49999-0008-55,50090-1941-00,50090-1941-02,52125-0034-02,52125-0054-02,52125-0150-02,52125-0366-02,52125-0522-55,52125-0562-08,52125-0619-01,52125-0619-52,52125-0712-
01,52125-0712-08,52125-0775-02,52125-0775-03,52125-0775-08,52125-0775-13,52125-0775-15,52125-0775-38,52125-0775-50,52125-0803-08,52125-0957-02,52125-0957-03,52125-0957-06,52125-0957-
13,52125-0957-49,52125-0957-53,52125-0957-55,52125-0957-58,54569-4728-00,54868-0365-00,54868-0365-01,54868-0365-02,54868-0365-03,54868-1185-00,54868-1185-01,54868-3896-00,54868-3896-
01,54868-3896-02,55566-8301-00,55566-8301-01,55566-8301-02,55566-8401-00,55566-8401-01,55566-8401-02,58118-0001-00,58118-0001-02,58118-0001-03,58118-0001-08,58118-9738-00,58118-9738-

© 2023 Bradley CJ, et al. *JAMA Health Forum.*

01,58118-9738-02,58118-9738-03,58118-9738-08,58118-9918-02,58177-0932-03,58177-0932-05,58177-0932-07,59630-0700-14,59630-0700-48,59630-0701-02,59630-0701-14,59630-0701-48,59630-0702-
14,59630-0702-48,60687-0132-11,60687-0132-21,61786-0049-02,61786-0049-03,61786-0049-08,61786-0049-13,61786-0049-15,61786-0049-38,61786-0049-50,61919-0365-05,61919-0365-21,62756-0093-
40,62756-0093-44,62756-0094-40,62756-0094-44,62756-0095-40,62756-0095-44,63629-4658-01,63629-5250-01,68084-0448-11,68084-0448-21,68084-0612-11,68084-0612-21,70529-0053-01,00046-1100-
51,00046-1100-52,00046-1100-81,00046-1100-91,00093-1125-89,00143-9597-21,00378-6920-78,42291-0024-12,51407-0181-12,64679-0021-01,64980-0418-12,69238-1165-07,00179-0244-70,00378-0640-
01,00378-0640-10,00378-0641-01,00378-0641-10,00615-0536-05,00615-0536-30,00615-0536-39,00615-3593-05,00615-3593-30,00615-3593-39,55700-0733-21,70518-1553-00,70518-1553-01,70518-1920-
00,76519-1224-03,62559-0670-30,69117-0003-01,69117-0003-04,59676-0600-12,59676-0600-56,59676-0600-99,00832-0285-00,69238-1250-01,42195-0151-10,76045-0109-10,68788-7267-00,68788-7267-
01,68788-7267-02,68788-7267-03,70362-0707-39,70121-1451-05,70121-1450-05,70121-1399-05,70529-0045-01,70529-0045-02,70529-0045-05,68788-7332-01,68788-7332-03,68788-7332-06,68788-7332-
09,70771-1374-03,68382-0383-06,70121-1463-02,59651-0180-30,59651-0180-90,70518-2020-00,69117-0004-01,70518-1541-00,68788-7343-02,76135-0009-01,76135-0010-01,76135-0011-01,76135-0005-
01,76135-0006-01,68788-7207-04,70518-1881-00,64980-0404-03,71329-0107-01,71329-0103-01,71329-0103-02,71329-0105-01,71329-0104-01,71329-0104-02,71329-0108-01,71329-0108-02,71329-0106-
01,71329-0106-02,70934-0259-21,70934-0259-30,70934-0259-42,70529-0048-01,70529-0048-02,70529-0048-03,70121-1654-01,70121-1651-01,70121-1651-05,70121-1652-01,70121-1653-01,70121-1655-
01,70121-1657-01,70121-1657-05,15014-0211-21,42195-0127-07,70518-1869-00,50090-3811-00,50742-0189-01,50742-0189-21,70518-1424-00,80425-0069-01,80425-0105-01,51662-1343-01,00046-1102-
51,00046-1102-52,00046-1102-81,00046-1102-91,65162-0240-03,51662-1261-01,51662-1262-01,50090-3466-00,00054-9817-25,00054-9817-29,00054-9818-25,00054-9818-29,00054-9828-25,00054-9828-
31,68788-7281-02,71335-1628-00,71335-1628-01,71335-1628-02,71335-1628-03,71335-1628-04,71335-1628-05,71335-1628-06,71335-1628-07,71335-1628-08,71335-1628-09,00009-7663-04,00054-0080-
13,00378-5001-93,00832-0595-30,47781-0108-30,54868-5261-00,59762-2858-01,00832-0086-00,00185-1125-01,00555-0870-01,49884-0753-05,49884-0753-13,54868-4628-00,55567-0150-06,55567-0150-
13,55567-0150-25,60429-0272-05,60429-0272-18,69097-0915-12,69097-0915-91,50090-2027-00,67979-0500-01,00009-0012-01,00009-0031-01,00009-0044-01,00115-1696-06,00115-1697-01,00115-1700-
01,00143-1252-01,00143-1252-30,00143-1253-01,00143-1253-30,00179-0056-70,00179-0058-70,00179-0155-70,00904-2674-60,16590-0990-30,16590-0990-60,16590-0990-90,42543-0140-50,42543-0141-
01,42543-0142-01,43063-0208-01,43063-0208-30,43063-0208-60,43063-0386-30,54505-0331-05,54505-0332-10,54505-0333-10,54868-1743-00,54868-1743-01,54868-1743-02,54868-1743-03,54868-1743-
04,54868-3924-00,59366-2807-02,59366-2817-03,59366-2827-03,59762-0073-01,59762-0074-01,59762-0075-01,60429-0262-01,63629-7241-01,63629-7241-02,64720-0331-05,64720-0332-10,64720-0333-
10,68084-0469-01,68084-0469-11,68084-0789-11,68084-0789-21,68084-0789-25,68084-0789-95,68084-0930-11,68084-0930-21,68084-0930-25,68084-0930-95,68151-2269-01,68151-4021-01,69189-0075-
01,69189-3320-01,00009-0005-01,00009-0013-05,00009-0013-06,00009-0016-12,00009-0825-01,00009-0900-13,00009-0900-20,00009-0912-05,00009-0920-03,00409-4856-05,00409-4856-15,54868-0605-
00,55154-3925-05,55154-3942-05,62559-0540-15,62559-0540-55,67457-0886-05,00004-0155-49,00004-0156-49,00004-0169-49,54868-0955-00,00555-1054-56,00555-1054-86,00555-1055-56,00555-1055-
86,00555-1056-86,00555-1057-56,00555-1057-86,10631-0115-31,10631-0116-31,10631-0117-31,10631-0118-31,10631-0133-31,10631-0134-31,54868-5039-00,54868-5041-00,54868-5043-00,54868-5305-
00,54868-6185-00,54868-6307-00,54868-6308-00,54868-6366-00,54868-6378-00,54868-6425-00,54868-6426-00,55111-0113-78,55111-0113-81,55111-0135-81,55111-0136-81,55111-0137-81,61748-0301-
01,61748-0301-11,61748-0301-13,61748-0302-01,61748-0302-11,61748-0302-13,61748-0303-01,61748-0303-11,61748-0303-13,61748-0304-01,61748-0304-11,61748-0304-13,69238-1017-03,69238-1174-
03,69238-1175-03,69238-1176-03,15054-0060-01,15054-0090-01,15054-0120-01,00054-0269-13,00078-0249-15,00093-7620-56,00179-0169-70,00527-1712-30,00603-4180-16,16729-0034-01,16729-0034-
10,16729-0034-15,16729-0034-16,16729-0034-17,16729-0034-43,24535-0801-01,24724-0030-01,24724-0030-03,42254-0243-30,42291-0373-90,42291-0374-90,51991-0759-10,51991-0759-33,54868-4151-
00,54868-6252-00,55111-0646-30,55111-0646-78,55111-0646-79,57884-2021-01,60505-3255-01,60505-3255-03,60505-3255-08,62175-0888-32,55154-4914-00,55289-0582-04,55289-0582-06,55289-0582-
10,55289-0582-21,55289-0582-28,55289-0903-10,55289-0903-12,55289-0903-20,60432-0466-00,60432-0466-08,61919-0269-21,61919-0269-55,63187-0383-05,63187-0383-24,63187-0383-30,63187-0383-
60,63187-0383-90,63629-2696-01,63629-3742-00,63629-3742-01,63629-3742-02,63629-3742-03,63629-3742-04,63629-3742-05,63629-3742-06,63629-3742-07,63629-3742-08,63629-3742-09,64679-0810-
08,64980-0509-24,66267-0067-12,66267-0067-21,66336-0479-40,66992-0015-01,67296-1090-01,68151-5026-04,68788-9938-01,68788-9938-03,68788-9938-05,68788-9939-00,68788-9939-01,68788-9939-
02,68788-9939-03,68850-0001-08,69189-4186-01,00517-4901-25,00517-4905-25,00517-4930-25,00641-0367-21,00641-0367-25,00641-6145-01,00641-6145-25,00641-6146-01,00641-6146-25,00703-3524-
01,00703-3524-03,14049-0910-02,46066-0223-04,46066-0505-02,49349-0917-41,50090-0414-00,50090-1715-00,50090-1922-00,50090-2363-00,52584-0165-05,52584-0420-10,52584-0421-00,52584-0422-
00,52584-0516-10,53225-3660-01,54569-3040-00,54569-4648-01,54569-4648-02,54868-0871-00,54868-0871-01,54868-0871-06,54868-6099-00,55150-0237-01,55150-0238-05,55150-0239-30,55154-2732-
05,55154-2732-08,55154-2733-05,55154-2733-08,55154-5118-05,55154-5118-08,55154-7075-05,55154-9364-05,55154-9364-08,55154-9371-05,55154-9387-05,61786-0372-02,63323-0165-01,63323-0165-
05,63323-0165-16,63323-0165-26,63323-0165-30,63323-0506-01,63323-0506-16,63323-0516-10,67457-0418-00,67457-0418-05,67457-0419-00,67457-0419-01,67457-0420-00,67457-0420-10,67457-0421-
00,67457-0421-30,67457-0422-00,67457-0422-54,67457-0423-00,67457-0423-12,67457-0483-00,67457-0483-10,67457-0484-00,67457-0484-30,68071-1866-01,70518-0621-00,76045-0106-10,55154-5707-
05,00469-0125-99,00179-0073-70,00179-0074-70,00179-0123-70,00339-6404-11,00378-1452-01,00378-1452-05,00378-1454-01,00378-1454-05,00378-1458-01,00378-1458-05,00378-1458-77,00430-0720-
24,00430-0721-24,00430-0722-24,00555-0899-02,00591-0487-01,00591-0487-05,00591-0488-01,00591-0488-05,00591-0528-01,00591-0528-05,10544-0069-30,10544-0070-30,16590-0052-71,16590-0278-
30,21695-0613-00,21695-0613-30,21695-0623-00,21695-0623-30,23155-0013-01,23155-0014-01,23155-0014-05,23155-0015-01,33261-0714-60,42806-0087-01,42806-0087-05,42806-0088-01,42806-0088-
05,42806-0089-01,42806-0089-05,42806-0089-09,43063-0201-01,43063-0201-30,43063-0201-90,43063-0446-01,43063-0446-30,43063-0446-90,43063-0651-30,43063-0706-30,43063-0706-90,43063-0717-
30,43063-0717-90,49999-0083-30,50090-0666-00,50090-0666-02,50090-0667-00,50090-1343-00,50090-2490-00,50090-2624-00,50268-0290-11,50268-0290-15,50268-0291-11,50268-0291-15,50268-0292-
11,50268-0292-15,51138-0037-30,51138-0050-30,51138-0051-30,51862-0332-01,51862-0333-01,51862-0333-05,51862-0334-01,51862-0334-05,54569-4907-00,54569-4907-02,54569-4908-00,54868-4030-
00,54868-4030-01,54868-4030-02,54868-4031-00,54868-4031-01,54868-4031-02,54868-4031-03,54868-4370-00,54868-4370-01,54868-4370-02,55154-6242-00,55154-6242-04,55154-6242-06,55289-0603-
07,55289-0603-21,55289-0603-30,55289-0603-90,55289-0761-01,55289-0761-30,55289-0761-90,55700-0458-30,55700-0458-90,55700-0504-90,60429-0327-01,60429-0328-01,60429-0328-05,60429-0329-
01,60429-0329-05,60429-0833-01,60429-0834-01,60429-0834-05,60429-0835-01,60429-0835-05,63187-0482-30,63187-0482-60,63187-0482-90,63187-0741-30,63187-0741-60,63187-0741-90,63629-2766-
01,63629-5208-01,63629-5208-02,63629-5208-03,63629-5208-04,63629-6467-01,64205-0048-30,64205-0048-90,64205-0488-30,64205-0488-90,66267-0092-30,66267-0092-90,66267-0093-30,66267-0093-
60,66267-0093-91,68071-3170-03,68071-3170-06,68071-3170-09,68071-3202-02,68071-3202-03,68071-3202-06,68071-3202-09,68788-9416-01,68788-9416-03,68788-9416-06,68788-9416-09,68788-9901-
01,68788-9901-03,68788-9901-06,68788-9901-09,69189-0487-01,69189-0886-01,69189-0899-01,00574-0870-05,00574-0872-05,42023-0110-01,42023-0111-01,42023-0112-01,42023-0134-01,42023-0135-
01,42023-0136-01,61570-0180-01,61570-0181-01,61570-0182-01,35356-0249-00,35356-0250-00,35356-0251-00,35356-0426-30,43353-0687-60,43353-0688-60,49999-0109-00,49999-0109-30,49999-0109-
90,50090-1853-03,53808-0770-01,54569-0811-03,54569-0812-00,54569-0812-05,54569-0813-01,54868-0451-00,54868-0451-02,54868-0451-06,54868-0452-02,54868-0453-00,54868-2702-00,54868-2702-
01,54868-4865-00,55154-0216-00,55154-0216-04,55154-0216-06,55154-0217-00,55154-0217-04,55154-0217-06,55154-0218-08,55154-0219-08,69189-1105-01,54868-5934-00,54868-5934-01,54868-6211-
00,54868-6211-01,54868-6212-00,54868-6212-01,61570-0072-01,61570-0073-01,61570-0074-01,61570-0075-50,69189-0078-01,00143-9750-01,00574-0821-05,00591-3221-26,67979-0501-40,58468-0030-
02,58468-1849-04,11399-0005-01,11399-0005-07,11399-0005-30,00555-0808-02,10370-0268-01,42291-0843-01,68462-0792-01,69189-0464-01,00003-0293-05,00003-0293-20,00003-0293-28,00003-0494-
20,00781-3243-75,00781-3245-70,00781-3245-72,00781-3245-75,21695-0360-01,21695-0360-10,49349-0946-41,49349-0947-41,50090-0256-00,50090-0295-01,50090-0819-00,50090-2360-00,53225-3610-

© 2023 Bradley CJ, et al. *JAMA Health Forum.*

01,54868-0234-00,54868-0235-00,54868-0235-01,54868-0235-02,54868-0284-00,55045-3248-01,55045-3248-04,63187-0661-01,68258-8903-05,68788-0636-01,70518-0075-00,00781-3084-75,54868-3344-00,00023-5902-04,00023-5904-12,00023-5906-23,52544-0092-76,52544-0153-02,52544-0154-02,52544-0156-02,52544-0188-76,52544-0189-76,55700-0072-21,55700-0072-30,55700-0072-60,55700-0072-90,55700-0203-10,55700-0203-14,55700-0203-15,55700-0203-18,55700-0203-20,55700-0203-21,55700-0203-30,55700-0208-15,55700-0208-20,55700-0208-21,55700-0208-30,55700-0208-40,55700-0208-48,55700-0208-60,55700-0209-15,55700-0209-20,55700-0209-21,55700-0209-30,55700-0209-42,55700-0209-48,58118-0018-01,58118-0018-02,58118-0018-03,58118-0018-08,58118-0027-08,58118-4728-00,58118-4728-03,58118-4728-08,58118-4728-09,58118-5338-00,58118-5338-01,58118-5338-03,58118-5338-08,58118-5442-08,59115-0139-01,59115-0139-10,59115-0140-01,59115-0140-10,59115-0141-01,59115-0141-05,59115-0141-10,59115-0141-55,59746-0171-06,59746-0171-10,59746-0172-06,59746-0172-10,59746-0173-06,59746-0173-09,59746-0173-10,59746-0175-06,59746-0175-09,59746-0175-10,60429-0130-01,60429-0130-10,60429-0131-01,60429-0131-05,60429-0131-10,60429-0132-01,60429-0132-05,60429-0132-10,60687-0122-01,60687-0122-11,60687-0134-01,60687-0134-11,60687-0145-01,60687-0145-11,60760-0629-15,60760-0629-21,60760-0629-30,60760-0629-60,61786-0633-08,61786-0869-05,61919-0373-21,61919-0373-30,61919-0404-21,61919-0404-30,61919-0995-07,63187-0037-05,63187-0037-08,63187-0037-09,63187-0037-10,63187-0037-20,63187-0037-21,63187-0037-30,63187-0037-40,63187-0037-42,63187-0066-05,63187-0066-06,63187-0066-08,63187-0066-09,63187-0066-12,63187-0066-15,63187-0066-20,63187-0066-21,63187-0066-24,63187-0066-30,63187-0066-40,63187-0066-42,63187-0085-05,63187-0085-06,63187-0085-09,63187-0085-10,63187-0085-15,63187-0085-20,63187-0085-21,63187-0085-24,63187-0085-30,63187-0243-05,63187-0243-08,63187-0243-10,63187-0243-15,63187-0243-20,63187-0243-21,63187-0243-30,63187-0243-40,63629-1579-00,63629-1579-01,63629-1579-02,63629-1579-03,63629-1579-04,63629-1579-05,63629-1579-06,63629-1579-07,63629-1579-08,63629-1579-09,63629-1587-00,63629-1587-01,63629-1587-02,63629-1587-03,63629-1587-04,63629-1587-05,63629-1587-06,63629-1587-07,63629-1587-08,63629-1587-09,63629-1605-00,63629-1605-01,63629-1605-02,63629-1605-03,63629-1605-04,63629-1605-05,63629-1605-06,63629-1605-07,63629-1605-08,63629-1605-09,63629-6621-01,63629-6621-02,63629-6621-03,63629-6621-04,63629-6621-05,63629-6621-06,63629-6621-07,63629-6621-08,63629-6621-09,63629-6658-01,63629-6659-01,63629-6659-02,63629-6659-03,63629-6659-04,63629-6659-05,63629-6659-06,63629-6660-01,63629-7388-01,63739-0518-10,63739-0519-10,63739-0520-10,63739-0588-10,64725-1473-01,66116-0485-21,66336-0058-15,66336-0058-90,66336-0094-15,66336-0094-21,66336-0219-30,66336-0428-30,66336-0428-60,66336-0428-90,67296-0247-01,67296-0247-02,67296-0247-03,67296-0247-05,67296-0247-06,67296-0600-01,67296-0600-02,67296-0600-03,67296-0602-01,67296-0602-02,67296-0974-01,67296-0974-02,67296-0974-05,67296-1032-01,67296-1032-03,67296-1032-04,67296-1194-03,67296-1194-05,67296-1203-01,68151-0820-08,68258-3013-01,68387-0240-10,68387-0240-25,68387-0241-15,68788-1473-01,68788-1473-02,68788-1473-03,68788-1473-04,68788-1473-05,68788-1473-06,68788-1473-08,68788-1473-09,68788-6414-02,68788-6440-01,68788-6440-02,68788-6440-03,68788-6440-04,68788-6440-05,68788-6440-06,68788-6440-07,68788-6440-08,68788-6440-09,68788-7372-01,68788-7372-02,68788-7372-03,68788-7372-04,68788-7372-08,68788-7372-09,68788-9177-01,68788-9177-02,68788-9177-03,68788-9177-04,68788-9177-08,68788-9177-09,68788-9178-01,68788-9178-02,68788-9178-03,68788-9178-04,68788-9178-05,68788-9178-06,68788-9178-07,68788-9178-08,68788-9178-09,68788-9179-01,68788-9179-02,68788-9179-03,68788-9179-04,68788-9179-06,68788-9179-07,68788-9179-08,68788-9179-09,68788-9309-01,68788-9309-02,68788-9309-03,68788-9309-04,68788-9309-05,68788-9309-06,68788-9309-07,68788-9309-08,68788-9309-09,68788-9503-01,68788-9503-02,68788-9503-03,68788-9503-04,68788-9503-06,68788-9503-07,68788-9503-08,68788-9503-09,68788-9513-00,68788-9513-01,68788-9513-02,68788-9513-03,68788-9513-04,68788-9513-06,68788-9513-07,68788-9513-08,68788-9513-09,68788-9551-02,69668-0120-20,70518-0205-00,70518-0305-00,70786-0172-01,70786-0172-04,70882-0117-21,70882-0117-30,75987-0020-01,75987-0020-02,75987-0020-70,75987-0021-01,75987-0021-02,75987-0021-70,75987-0022-01,75987-0022-02,75987-0022-70,76237-0227-30,76237-0228-30,00093-7290-01,00093-7290-10,00093-7290-56,00179-0151-70,00179-0212-70,00179-0220-70,00591-2367-01,00591-2367-10,00591-2367-30,42291-0726-10,42291-0726-90,43353-0253-60,43598-0505-01,43598-0505-10,43598-0505-30,49999-0458-30,50228-0306-20,50228-0306-30,52343-0137-01,54868-4170-00,54868-4170-01,55289-0266-30,65162-0057-03,65162-0057-09,65162-0057-10,65162-0057-11,65162-0057-33,65162-0057-50,65862-0709-01,65862-0709-03,65862-0709-10,65862-0709-22,65862-0709-30,66993-0417-02,66993-0417-30,66993-0417-88,66993-0661-02,66993-0661-30,66993-0661-88,69189-0418-01,69189-0678-01,69189-2367-01,00093-0782-01,00093-0782-05,00093-0782-10,00093-0782-56,00093-0784-05,00093-0784-06,00093-0784-10,00093-0784-86,00179-1952-01,00179-1952-30,00179-1952-50,00179-1952-60,00179-1952-90,00310-0600-30,00310-0604-60,00555-0446-05,00555-0446-09,00555-0446-63,00555-0904-01,00555-0904-05,00555-0904-14,00591-2232-18,00591-2232-60,00591-2233-19,00591-2233-30,00591-2472-18,00591-2472-60,00591-2473-19,00591-2473-30,13632-0123-01,50090-0485-01,50090-0942-00,50090-1998-01,50090-2533-01,51862-0446-01,51862-0446-05,51862-0446-10,51862-0446-30,51862-0447-05,51862-0447-10,51862-0447-18,51862-0447-60,51862-0449-18,51862-0449-60,51862-0450-30,51862-0450-90,54569-3765-01,54569-5716-00,54868-3004-01,54868-3004-02,54868-3004-03,54868-3004-04,54868-3004-05,54868-4287-00,54868-4287-01,54868-4287-02,54868-4287-03,54868-4287-04,60429-0909-60,60429-0910-30,60505-3035-03,60505-3035-05,60505-3035-06,60505-3035-07,60505-3036-01,60505-3036-03,60505-3036-07,68084-0924-11,68084-0924-21,68084-0935-11,68084-0935-21,64205-0077-10,64205-0087-30,64205-0087-90,66267-0140-10,66267-0140-30,66336-0603-20,67296-0396-01,68071-2175-03,68084-0553-11,68084-0553-21,68788-7401-01,68788-7401-03,68788-9899-01,68788-9899-03,69189-3740-01,69189-3741-01,00015-0595-01,00015-0596-41,00054-4603-25,00054-4604-25,00054-8603-25,00054-8604-25,00182-1863-89,00182-1864-00,00182-1864-89,00615-3570-31,00615-3570-39,00904-3571-61,49884-0289-01,49884-0290-01,49884-0290-04,49884-0290-05,51079-0434-01,51079-0434-20,51079-0435-01,51079-0435-20,53808-0614-01,54868-1629-00,54868-1629-01,54868-1629-02,54868-1629-03,55154-0734-00,55154-0885-03,55154-5390-00,55154-5516-00,60429-0433-01,60429-0433-05,63739-0165-10,69189-0608-01,00009-0020-01,00009-0022-01,00009-0073-01,00009-0176-01,00009-3547-01,00179-0124-70,00555-0301-02,00555-0301-04,00555-0301-38,00591-0790-01,00591-0790-21,00781-5022-01,00781-5022-07,00781-5022-10,00904-6574-61,16590-0149-21,16590-0879-21,21695-0080-21,33261-0335-21,35356-0763-21,42549-0522-21,49349-0564-16,49349-0750-05,49349-0884-05,49349-0957-05,49349-0969-05,49999-0153-21,49999-0153-30,49999-0153-60,49999-0153-90,50090-0092-00,50090-0221-00,50090-1885-00,50090-2638-00,51991-0188-01,51991-0188-31,54569-0327-00,54569-1036-00,54569-8856-00,54771-3547-01,54868-0776-01,54868-2913-00,54868-2913-01,54868-2913-02,54868-2913-03,54868-4952-00,54868-4952-01,54868-6624-01,55045-1259-09,55289-0649-30,59746-0001-03,59746-0001-06,59746-0002-04,59746-0002-06,59746-0003-14,59746-0015-04,59762-0049-01,59762-0050-01,59762-0051-01,59762-3327-01,59762-3327-02,59762-4440-02,59762-4440-03,61919-0149-21,63187-0160-21,63187-0802-21,63629-3910-01,63739-0133-10,63874-0413-21,66267-0961-21,68001-0005-01,68084-0149-01,68084-0149-11,68387-0170-01,68788-4593-02,68788-9218-02,69189-4593-01,70121-1000-05,70121-1001-05,70786-0001-01,00009-0274-01,00703-0031-01,00703-0031-04,00703-0043-01,00703-0045-01,00703-0051-01,00703-0051-04,00703-0063-01,00781-3131-71,00781-3131-95,00781-3132-71,00781-3132-95,00781-3136-70,00781-3136-92,00781-3137-75,00781-3137-95,21695-0849-10,21695-0850-05,21695-0952-05,50090-0312-01,50090-0436-00,50090-0556-00,50090-1823-00,50090-2098-00,53217-0123-01,53225-3650-01,54868-0590-00,54868-1994-00,55045-3243-05,55154-3929-05,55154-3932-05,61786-0027-57,63187-0474-01,70518-0516-00,00009-0003-02,00009-0018-20,00009-0113-12,00009-0698-01,00009-0758-01,00009-0765-02,00009-0796-01,00009-3389-01,00409-3218-05,00409-3218-15,25021-0807-05,25021-0808-10,25021-0810-30,49349-0840-41,50090-0271-00,50090-0271-01,50090-2253-00,52125-0144-01,52125-0515-01,52584-0039-30,52584-0047-25,52584-0113-12,52584-0190-09,54569-1555-00,54569-1555-01,54868-0768-00,54868-3623-00,54868-3637-00,54868-3637-01,54868-6199-00,55150-0262-03,55150-0263-03,55150-0264-20,55150-0265-50,55154-3914-05,55154-3915-05,55154-3939-05,55154-3940-05,55154-3940-08,55154-3941-05,55154-3941-08,55154-3944-05,55154-9383-05,55154-9557-05,55390-0209-10,55390-0210-10,55390-0218-01,55390-0219-01,55390-0258-01,55390-0259-01,61699-0047-02,63323-0255-03,63323-0258-03,63323-0265-30,70121-1000-01,70121-1001-01,70385-2016-01,70518-0503-00,00115-1408-01,00115-1408-03,00115-1408-08,00115-7037-01,21695-0765-48,24236-0107-03,24236-0205-20,24236-0405-02,33261-0129-00,33261-0129-10,33261-0129-12,33261-0129-15,33261-0129-20,33261-0129-21,33261-0129-27,33261-0129-30,33261-0129-40,33261-0129-42,33261-0129-48,33261-0129-50,33261-0129-60,33261-0129-90,33261-0351-00,33261-0351-10,33261-0351-12,33261-0351-15,33261-0351-20,33261-0351-21,33261-0351-27,33261-0351-30,33261-0351-40,33261-0351-42,33261-0351-48,33261-0351-50,33261-0351-60,33261-0351-90,33261-0352-00,33261-0352-10,33261-0352-12,33261-0352-15,33261-0352-20,33261-0352-21,33261-0352-27,33261-0352-30,33261-0352-40,33261-0352-42,33261-0352-48,33261-0352-50,33261-0352-

© 2023 Bradley CJ, et al. *JAMA Health Forum.*

60,33261-0352-90,33261-0780-01,35356-0673-20,35356-0673-21,35356-0673-30,35356-0673-40,35356-0673-42,35356-0673-60,35356-0673-90,35356-0674-00,35356-0674-10,35356-0674-15,35356-0674-
18,35356-0674-20,35356-0674-21,35356-0674-30,35356-0674-60,35356-0674-90,35356-0677-12,35356-0677-14,35356-0677-15,35356-0677-18,35356-0677-20,35356-0677-21,35356-0677-28,35356-0677-
30,35356-0677-40,35356-0677-48,35356-0677-60,35356-0677-90,35356-0818-10,35356-0818-15,35356-0818-18,35356-0818-20,35356-0818-21,35356-0818-30,35356-0819-15,35356-0819-20,35356-0819-
21,35356-0819-30,35356-0819-40,35356-0819-42,42549-0647-14,43063-0426-10,43063-0426-12,43063-0426-20,43063-0426-21,43063-0426-28,43063-0426-30,43063-0426-40,43063-0426-42,43063-0426-
50,43063-0426-60,43063-0472-05,43063-0472-06,43063-0472-07,43063-0472-09,43063-0472-10,43063-0472-12,43063-0472-14,43063-0472-15,43063-0472-20,43063-0472-21,43063-0472-25,43063-0472-
30,43063-0590-05,43063-0590-06,43063-0590-09,43063-0590-10,43063-0590-12,43063-0590-14,43063-0590-15,43063-0590-18,43063-0590-20,43063-0590-21,43063-0590-25,43063-0590-30,43063-0608-
21,43063-0610-21,43063-0643-21,43063-0643-55,43063-0644-10,43063-0644-15,43063-0644-20,43063-0644-21,43063-0644-30,43063-0644-40,43063-0644-42,43063-0644-50,43063-0644-60,43063-0703-
05,43063-0703-06,43063-0703-09,43063-0703-10,43063-0703-12,43063-0703-14,43063-0703-15,43063-0703-18,43063-0703-20,43063-0703-21,43063-0703-25,43063-0703-30,43353-0657-60,43353-0819-
60,45865-0638-21,45865-0670-40,48433-0321-01,48433-0321-10,49349-0059-02,49349-0059-24,49999-0028-12,49999-0028-14,49999-0028-15,49999-0028-20,49999-0028-21,49999-0028-30,49999-0028-
40,49999-0028-48,49999-0028-60,49999-0028-65,49999-0110-00,49999-0110-06,49999-0110-07,49999-0110-10,49999-0110-15,49999-0110-18,49999-0110-20,49999-0110-21,49999-0110-30,50090-0093-
00,50090-0094-04,50090-0095-00,50090-0095-01,50090-0095-03,50090-0095-04,50090-0095-07,50090-0096-04,50090-0097-00,50090-0097-01,50090-0097-02,50090-0097-04,50090-0097-05,50090-0097-
07,50090-0097-08,50090-0098-00,50090-0098-01,50090-0099-01,50090-0099-02,50090-0099-03,50090-0099-05,50090-0099-09,50090-0100-00,50090-0100-01,50090-0100-02,50090-0100-05,50090-0100-
06,50090-0100-07,50090-0101-00,50090-0102-00,50090-0439-00,50090-1001-00,50090-1002-00,50090-1455-00,50090-1989-01,50090-1989-02,50090-1989-03,50090-1989-05,50090-1989-09,50090-1990-
00,50090-1990-01,50090-1990-02,50090-1990-05,50090-1990-07,50090-1991-00,50090-2136-00,50090-2402-00,50090-2402-01,50090-2402-03,50090-2402-04,50090-2402-07,50090-2403-04,50090-2532-
00,50090-2532-01,50090-2532-02,50090-2532-04,50090-2532-05,50090-2532-07,50090-2532-08,50090-2608-00,50090-2608-01,50090-2822-00,50436-1720-01,50436-1720-04,50436-1720-05,50436-1750-
01,50436-1750-04,50436-4325-01,50436-4325-02,50436-4326-02,51138-0143-15,51138-0143-30,51138-0144-30,51138-0145-15,51138-0145-20,51138-0145-30,51138-0146-15,51138-0146-20,51138-0146-
21,51138-0146-28,51138-0146-36,51138-0146-39,51138-0146-42,51138-0146-50,51138-0147-10,51138-0147-12,51138-0147-14,51138-0147-15,51138-0147-20,51138-0147-21,51138-0147-30,51138-0154-
15,51138-0154-20,51138-0154-30,51138-0155-15,51138-0155-20,51138-0155-21,51138-0155-28,51138-0155-36,51138-0155-39,51138-0155-42,51138-0155-50,51138-0156-10,51138-0156-12,51138-0156-
14,51138-0156-15,51138-0156-20,51138-0156-21,51138-0156-30,51655-0320-18,51655-0320-20,51655-0320-21,51655-0320-30,51655-0320-53,51655-0320-54,51655-0410-20,51655-0410-49,52125-0255-
05,52125-0555-02,52125-0996-03,52959-0126-00,52959-0126-05,52959-0126-07,52959-0126-10,52959-0126-12,52959-0126-14,52959-0126-15,52959-0126-18,52959-0126-20,52959-0126-21,52959-0126-
25,52959-0126-30,52959-0126-37,52959-0126-40,52959-0126-42,52959-0126-44,52959-0126-45,52959-0126-50,52959-0126-60,52959-0126-70,52959-0127-07,52959-0127-10,52959-0127-11,52959-0127-
12,52959-0127-15,52959-0127-18,52959-0127-20,52959-0127-21,52959-0127-25,52959-0127-30,52959-0127-37,52959-0127-42,53217-0255-10,53217-0255-12,53217-0255-14,53217-0255-20,53217-0255-
21,53217-0255-24,53217-0255-27,53217-0255-30,53217-0255-40,53217-0255-42,53217-0255-48,53217-0255-50,53217-0255-60,53217-0255-90,53217-0288-07,53217-0288-10,53217-0288-12,53217-0288-
14,53217-0288-15,53217-0288-18,53217-0288-20,53217-0288-21,53217-0288-30,53217-0288-60,53217-0292-00,53217-0292-12,53217-0292-15,53217-0292-21,53217-0292-30,53217-0292-40,53217-0292-
50,53217-0292-60,53217-0292-90,53489-0138-01,53489-0138-05,53489-0138-10,53489-0138-50,53489-0138-60,53489-0139-01,53489-0139-05,53489-0139-10,53489-0140-01,53489-0140-02,53489-0140-
05,53489-0140-10,53808-0399-01,53808-0540-01,53808-0542-01,53808-0543-01,53808-0560-01,54569-0330-00,54569-0330-01,54569-0330-03,54569-0330-04,54569-0330-07,54569-0331-00,54569-0331-
01,54569-0331-02,54569-0331-04,54569-0331-05,54569-0331-07,54569-0331-08,54569-0332-01,54569-0332-02,54569-0332-03,54569-0332-05,54569-0332-09,54569-0333-00,54569-3043-00,54569-3043-
01,54569-3043-02,54569-3043-05,54569-3043-06,54569-3043-07,54569-3302-00,54569-3302-01,54569-3413-00,54569-4026-04,54569-5840-00,54868-0258-00,54868-0258-01,54868-0258-02,54868-0258-
03,54868-0258-04,54868-0258-05,54868-0258-06,54868-0258-07,54868-0258-08,54868-0258-09,54868-0836-00,54868-0836-01,54868-0836-02,54868-0836-03,54868-0836-04,54868-0836-05,54868-0836-
06,54868-0836-07,54868-0836-08,54868-0836-09,54868-0908-00,54868-0908-01,54868-0908-02,54868-0908-03,54868-0908-04,54868-0908-05,54868-0908-06,54868-0923-01,54868-1119-00,54868-1119-
01,54868-1119-02,54868-1119-03,54868-1119-04,54868-1119-05,54868-1183-00,54868-1183-01,54868-1183-02,54868-1183-03,54868-1183-04,54868-1183-06,54868-1183-07,54868-1183-08,54868-1183-
09,54868-4095-00,54868-4096-00,54868-5213-00,54868-5230-00,55154-4926-00,55154-4938-00,55154-4946-00,55154-4949-00,55154-4949-09,55154-4950-00,55154-4954-09,55154-4965-09,55289-0330-
05,55289-0330-07,55289-0330-10,55289-0352-05,55289-0352-07,55289-0352-09,55289-0352-10,55289-0352-12,55289-0352-14,55289-0352-15,55289-0352-20,55289-0352-21,55289-0352-30,55289-0373-
01,55289-0373-21,55289-0373-30,55289-0373-36,55289-0373-42,55289-0373-46,55289-0373-55,55289-0373-60,55289-0373-72,55289-0438-15,55289-0438-20,55289-0438-21,55289-0438-30,55289-0438-
36,55289-0438-38,55289-0438-40,55289-0438-42,55289-0438-50,55289-0438-60,55695-0013-00,55695-0014-00,55695-0015-00,55695-0024-00,55700-0049-21,55700-0072-15,55700-0072-18,55700-0072-
20,00093-6118-16,00093-6118-87,12634-0686-04,16477-0505-01,16477-0505-21,16477-0505-48,16590-0291-37,21695-0365-08,21695-0365-16,33261-0475-01,50383-0042-24,50383-0042-48,53217-0193-
01,54868-4748-00,54868-4749-00,54868-4749-01,58177-0910-05,58177-0910-07,60505-0361-00,60505-0362-00,60505-0362-01,61919-0291-37,63187-0466-64,63629-1862-01,66116-0603-24,68258-8987-
02,68788-9096-02,68788-9096-04,68788-9545-04,51672-1338-03,51672-1338-05,51672-1338-06,51672-1338-08,00121-0759-08,00121-0773-08,00121-0777-08,00574-0148-04,00603-1572-54,00603-1572-
58,13925-0166-04,13925-0501-04,16477-0510-08,23594-0510-08,23594-0915-08,42254-0102-08,42799-0812-01,42799-0813-01,50090-0955-00,50090-1411-00,50090-1582-00,50383-0040-04,53014-0250-
01,54868-0821-00,54868-5242-00,54868-5242-01,55154-3276-04,55154-3276-05,55154-3276-06,58177-0931-03,59630-0710-08,59630-0710-10,60432-0212-08,60432-0212-16,63187-0215-64,63717-0915-
08,65162-0667-76,65162-0667-88,65162-0667-90,65580-0251-01,67296-1505-06,68791-0100-04,00054-0017-20,00054-0017-25,00054-0017-29,00054-0018-20,00054-0018-25,00054-0018-29,00054-0019-
20,00054-0019-25,00054-3721-44,00054-4728-25,00054-4728-31,00054-4741-25,00054-4741-31,00054-4742-25,00054-8722-16,00054-8724-25,00054-8739-25,00054-8740-25,00143-1425-01,00143-1425-
05,00143-1425-10,00143-9741-01,00143-9741-05,00143-9741-10,00440-8165-12,00440-8167-01,00440-8167-05,00440-8167-10,00440-8167-15,00463-6141-05,00463-6155-10,00603-5338-15,00603-5338-
21,00603-5338-28,00603-5338-31,00603-5338-32,00615-2513-39,00615-6516-39,10544-0045-15,10544-0045-20,10544-0046-20,10544-0046-30,10544-0046-42,10544-0048-20,10544-0048-42,10544-0093-
42,10544-0125-10,10544-0125-15,10544-0125-30,10544-0274-21,10544-0274-30,10544-0473-20,10544-0508-20,10544-0508-30,10544-0508-42,10544-0509-07,10544-0509-10,10544-0509-12,10544-0509-
15,10544-0509-20,10544-0509-30,10544-0538-21,10544-0538-30,10544-0913-30,10544-0914-20,10544-0914-21,10544-0915-10,10544-0915-30,10768-7085-01,10768-7283-03,10768-7283-04,10768-7733-
03,10768-7733-04,12634-0184-00,12634-0184-01,12634-0184-09,12634-0184-40,12634-0184-42,12634-0184-50,12634-0184-52,12634-0184-54,12634-0184-57,12634-0184-59,12634-0184-60,12634-0184-
61,12634-0184-63,12634-0184-66,12634-0184-67,12634-0184-69,12634-0184-71,12634-0184-74,12634-0184-78,12634-0184-79,12634-0184-80,12634-0184-81,12634-0184-82,12634-0184-85,12634-0184-
91,12634-0184-94,12634-0184-95,12634-0184-96,12634-0188-00,12634-0188-01,12634-0188-52,12634-0188-54,12634-0188-55,12634-0188-57,12634-0188-71,12634-0188-79,12634-0188-80,12634-0188-
81,12634-0188-82,12634-0188-84,12634-0188-85,12634-0188-91,16590-0326-10,16590-0326-15,16590-0326-20,16590-0326-21,16590-0326-30,16590-0365-21,16590-0373-21,16590-0373-30,16590-0373-
48,16590-0373-60,16590-0373-90,16590-0624-21,16590-0624-48,17856-3722-01,21695-0305-30,21695-0306-20,21695-0306-21,21695-0306-28,21695-0306-30,21695-0306-36,21695-0306-39,21695-0306-
42,21695-0306-50,21695-0307-10,21695-0307-12,21695-0307-14,21695-0307-15,21695-0307-20,21695-0307-21,21695-0307-30,21695-0580-05,21695-0580-14,21695-0764-21,21695-0765-21,00187-0901-
01,00187-0902-01,76346-0073-01,76346-0073-02,24987-0111-14,24987-0111-15,59212-0111-10,59212-0111-14,62559-0173-31,63323-0365-01,63323-0365-04,63323-0376-01,63323-0376-04,63323-0377-
01,63323-0377-04,63323-0378-05,63323-0379-05,00078-0184-25,00078-0340-61,00078-0341-61,00078-0342-61,00078-0646-81,00078-0648-81,00078-0811-81,00078-0818-81,00078-0825-81,00078-9342-

© 2023 Bradley CJ, et al. *JAMA Health Forum.*

61,00641-6174-01,00641-6174-10,00641-6175-01,00641-6175-10,00641-6176-01,00641-6176-10,00641-6177-01,00641-6178-01,00703-3301-01,00703-3301-04,00703-3311-01,00703-3311-04,00703-3321-
01,00703-3321-04,00703-3321-91,00703-3321-94,00703-3333-01,00703-3343-01,00781-3164-75,00781-3165-75,00781-3166-71,00781-3166-95,00781-3167-71,00781-3167-95,00781-3168-71,00781-3168-
95,00781-9164-75,00781-9165-75,25021-0451-01,25021-0452-01,25021-0453-01,25021-0454-05,25021-0455-05,55390-0160-10,55390-0161-10,55390-0162-10,55390-0163-01,55390-0164-01,55390-0375-
10,55390-0376-10,55390-0377-01,55648-0632-01,55648-0633-01,55648-0633-02,55648-0634-01,55648-0635-01,55648-0635-02,55648-0636-01,55648-0636-02,62756-0348-44,62756-0349-44,62756-0350-
40,62756-0351-44,62756-0352-40,64679-0632-01,64679-0633-01,64679-0633-02,64679-0634-01,64679-0635-01,64679-0635-02,64679-0636-01,64679-0636-02,67457-0239-00,67457-0239-01,67457-0245-
00,67457-0245-01,67457-0246-00,67457-0246-01,62756-0511-08,62756-0511-18,62756-0511-83,62756-0511-88,63323-0772-30,65841-0744-06,65841-0744-10,65841-0744-30,68084-0803-11,68084-0803-
21,68382-0363-06,68382-0363-10,68382-0363-30,69189-7620-01,00024-0222-05,00024-0605-45,00024-0610-30,00024-0793-75,00074-3680-01,00781-4003-32,41616-0936-40,47335-0936-40,49884-0368-
26,62935-0222-05,62935-0223-05,62935-0302-30,62935-0303-30,62935-0452-45,62935-0453-45,62935-0752-75,62935-0753-75,00009-0286-03,00009-0626-01,10544-0001-30,10544-0062-10,21695-0896-
10,21695-0896-20,21695-0896-30,33261-0534-05,33261-0534-10,33261-0534-14,33261-0534-30,33261-0609-30,33261-0740-20,33261-0740-28,33261-0740-30,43063-0438-05,43063-0438-07,43063-0438-
10,43063-0438-13,43063-0438-30,43063-0438-40,43063-0438-42,43063-0438-50,43063-0438-90,50090-0166-00,50090-0166-02,54569-0809-00,54569-0809-02,54569-3806-02,54569-3807-01,54868-0109-
00,54868-0109-01,54868-0109-02,54868-0109-03,54868-0109-05,54868-0109-06,54868-0109-07,54868-0109-08,54868-0290-00,54868-0290-02,54868-0290-03,54868-0290-04,54868-1010-01,54868-1010-
03,54868-1010-04,54868-2984-00,54868-2984-02,54868-2984-03,54868-2985-00,54868-2985-01,54868-2985-02,54868-2985-03,54868-3348-01,54868-3613-00,54868-4100-00,54868-4100-01,55154-0533-
00,55154-0533-04,55154-0533-06,55289-0160-05,55289-0160-07,55289-0160-10,55289-0160-13,55289-0160-30,55289-0160-40,55289-0160-42,55289-0160-50,55289-0816-30,55289-0908-30,55289-0908-
42,59762-3740-01,59762-3740-04,59762-3740-05,59762-3741-01,59762-3741-04,59762-3742-01,59762-3742-02,59762-3742-03,59762-3742-06,59762-3742-07,59762-3742-08,60687-0105-11,60687-0105-
21,63629-2613-01,63629-2613-02,63629-2613-03,63629-2613-04,63629-6812-01,63629-6812-02,55154-4901-00,60687-0582-01,70934-0350-30,64380-0972-06,76420-0110-50,57582-0101-01,57582-0101-
02,68071-5221-01,68788-7833-02,76420-0193-21,69306-0400-21,70518-2864-00,70518-2942-00,70934-0605-10,70934-0605-30,63187-0382-10,63187-0382-20,63187-0382-30,63187-0382-40,63187-0382-
60,63187-0382-90,63629-2612-01,63629-2612-02,63629-2612-03,63629-2612-04,63629-2612-05,63629-2612-06,63629-2612-07,63629-2614-01,63629-2614-02,63629-2614-03,63629-2614-04,63629-2614-
05,50090-0491-00,50090-0491-01,72189-0008-21,70518-0443-00,70518-0443-01,50090-0490-00,50090-0490-02,70518-3070-00,71329-0302-25,71329-0301-25,71329-0303-01,71329-0305-01,71329-0304-
01,60760-0373-21,54348-0506-10,54348-0506-20,00615-8391-05,00615-8391-30,00615-8391-39,50436-4324-01,50436-4324-02,50436-4324-03,70934-0307-10,70934-0307-15,70934-0307-18,70934-0307-
20,70934-0307-21,70934-0307-30,70934-0291-12,70934-0291-15,70934-0291-20,70934-0291-21,70934-0291-30,70934-0291-40,70934-0291-42,70934-0290-21,70934-0290-30,70518-1105-00,70518-1105-
01,70518-1105-02,70518-1105-03,70518-1105-04,70934-0137-05,70934-0137-07,51655-0355-26,67296-1704-02,70518-2138-00,70518-2138-01,70518-2172-00,70934-0365-30,70518-2516-00,70934-0831-
10,70934-0831-12,70934-0831-15,70934-0831-21,70934-0831-30,70934-0831-42,50090-0655-00,50090-0655-01,50090-0655-02,50090-2522-01,59212-0701-02,59212-0701-12,59212-0701-48,50090-2522-
00,59212-0700-12,59212-0700-48,59212-0702-12,59212-0702-48,50436-0759-01,50436-0759-02,66993-0844-35,66993-0844-62,66993-0845-35,66993-0845-62,66993-0846-35,66993-0846-62,44523-0182-
08,68788-7708-02,42291-0771-01,42291-0771-50,50090-4784-01,50090-4784-02,50090-4784-03,50090-4784-05,50090-4784-09,50090-4785-00,50090-4785-01,50090-4785-02,50090-4785-05,50090-4785-
07,52959-0220-00,52959-0220-05,52959-0220-06,52959-0220-10,52959-0220-12,52959-0220-20,52959-0220-21,52959-0220-23,52959-0220-30,52959-0220-36,52959-0220-40,52959-0220-60,52959-0220-
75,71335-1737-01,71335-1737-02,71335-1736-01,71335-1736-02,71335-1736-03,71335-1736-04,71335-1736-05,71335-1736-06,71335-1525-00,71335-1525-01,71335-1525-02,71335-1525-03,71335-1525-
04,71335-1525-05,71335-1525-06,71335-1525-07,71335-1525-08,71335-1525-09,71335-1524-00,71335-1524-01,71335-1524-02,71335-1524-03,71335-1524-04,71335-1524-05,71335-1524-06,71335-1524-
07,71335-1524-08,71335-1524-09,71335-1516-01,71335-1516-02,71335-1516-03,71335-1516-04,71335-1516-05,71335-1516-06,71335-1516-07,71205-0460-05,71205-0460-07,71205-0460-30,71205-0460-
60,71205-0460-90,71205-0421-10,71205-0421-18,71205-0421-20,71205-0421-30,71205-0421-40,71205-0421-42,71205-0421-60,71205-0421-90,71205-0407-10,71205-0407-15,71205-0407-18,71205-0407-
20,71205-0407-21,71205-0407-30,71205-0407-60,71205-0407-90,71205-0403-05,71205-0403-07,71205-0403-10,71205-0403-12,71205-0403-30,71205-0403-60,71205-0403-90,70954-0060-10,70954-0060-
20,70954-0060-30,70954-0059-10,70954-0059-20,70954-0059-30,70954-0059-40,70954-0058-10,70954-0058-20,70954-0058-30,70954-0058-40,70934-0580-10,70934-0580-15,70934-0580-20,70934-0580-
21,70934-0580-30,70934-0580-40,70934-0580-42,70934-0471-10,70934-0471-12,70934-0471-15,70934-0471-18,70934-0471-20,70934-0471-21,70934-0471-30,70934-0471-40,70934-0218-10,70934-0218-
15,70934-0218-20,70934-0218-21,70934-0218-30,70934-0095-20,70934-0095-21,70934-0095-30,70934-0095-42,70934-0096-10,70934-0096-15,70934-0096-20,70934-0096-30,70518-2916-00,70518-2916-
01,70518-2916-02,70518-2916-03,70518-0242-00,70518-0242-01,70518-0242-02,70518-0242-03,70518-0242-04,70518-0242-05,70518-0073-00,70518-0073-01,70518-0073-02,70518-0073-03,70518-0073-
04,70518-0073-05,70518-0073-06,70518-0073-07,70518-0073-08,61786-0538-02,61786-0538-03,61786-0538-55,61786-0538-58,63187-0020-10,63187-0020-15,63187-0020-21,63187-0020-30,63187-0020-
36,61919-0342-05,61919-0342-10,61919-0342-12,61919-0342-14,61919-0342-20,61919-0342-21,61919-0342-30,61919-0235-10,61919-0235-15,61919-0235-21,61919-0235-30,61919-0235-40,61919-0235-
42,63187-0300-05,63187-0300-06,63187-0300-08,63187-0300-09,63187-0300-10,63187-0300-12,63187-0300-15,63187-0300-18,63187-0300-20,63187-0300-21,63187-0300-24,63187-0300-27,63187-0300-
28,63187-0300-30,63187-0300-36,63187-0300-40,63187-0300-42,63187-0807-05,63187-0807-06,63187-0807-07,63187-0807-08,63187-0807-09,63187-0807-10,63187-0807-12,63187-0807-14,63187-0807-
15,63187-0807-18,63187-0807-20,63187-0807-21,63187-0807-24,63187-0807-30,63187-0807-40,63187-0807-42,63629-4562-01,63629-4562-02,63629-4562-03,63629-4562-04,63629-4562-05,63629-4562-
06,63629-4562-07,50090-2789-01,50090-2789-02,50090-2789-03,50090-2789-05,50090-2789-09,50090-2804-00,50090-2804-01,50090-2804-02,50090-2804-05,50090-2804-07,55700-0728-20,55700-0728-
21,61919-0321-21,61919-0321-30,61919-0321-42,43063-0097-06,43063-0097-09,70518-1561-00,70518-1561-01,70518-1854-00,70518-1854-01,70518-0473-00,70518-0473-01,70518-0473-02,70518-0473-
03,70518-0632-00,70518-0632-01,67296-0140-01,67296-0140-02,67296-0140-03,67296-0140-09,61919-0326-10,61919-0326-15,61919-0326-20,61919-0326-21,61919-0326-23,68788-7637-01,68788-7637-
02,68788-7637-03,68788-7637-04,68788-7637-06,68788-7637-07,68788-7637-08,68788-7637-09,68788-7752-00,68788-7752-01,68788-7752-02,68788-7752-03,68788-7752-04,68788-7752-05,68788-7752-
06,68788-7752-08,68788-7752-09,70518-2314-00,70518-2314-01,70518-2115-00,70518-2115-01,42291-0727-10,51655-0208-20,50090-5453-00,42708-0136-21,42291-0770-50,42291-0768-01,42291-0769-
01,60760-0615-18,60760-0715-21,67296-1755-01,67296-1755-02,67296-1755-07,67296-1331-07,67296-1419-03,68071-2209-01,68071-2304-01,68071-5242-02,68071-5195-02,68071-5233-01,68071-5196-
01,67296-1767-01,67296-1767-03,68788-7692-01,68788-7692-02,68788-7692-03,68788-7692-04,68788-7692-08,68788-7692-09,70518-2675-00,70518-2704-00,70518-2703-00,70518-2486-00,70518-2676-
00,70518-2676-01,70518-2555-00,70934-0543-05,70934-0645-30,70934-0699-21,70934-0647-30,70934-0674-12,70954-0061-10,70954-0057-10,00009-0050-02,00009-0050-11,00009-0280-02,00009-0280-
03,00009-0280-24,00009-0280-25,00009-0280-51,00009-0280-52,00009-0306-02,00009-0306-12,00009-0306-24,00009-3073-01,00009-3073-03,00009-3073-22,00009-3073-23,67157-0111-05,67157-0111-
25,70121-1049-02,70121-1049-05,70518-2459-00,70518-3071-00,51662-1429-01,52584-0238-05,52584-0239-30,70121-1574-01,70121-1574-05,10631-0002-31,10631-0003-31,10631-0004-31,10631-0005-
31,10631-0006-31,10631-0007-31,57664-0022-97,69665-0210-01,69665-0310-01,72143-0231-30,72143-0233-30,72143-0234-30,70121-1573-01,70121-1573-05,70518-2894-00,70518-2484-00,55150-0311-
00,55150-0311-01,55150-0311-05,71205-0451-01,72603-0108-01,72603-0202-01,72603-0401-01,70112-0555-02,16714-0088-01,16714-0090-01,16714-0472-01,16714-0089-01,16714-0473-01,16714-0088-
25,16714-0472-25,71205-0537-06,71205-0537-07,71205-0537-12,71205-0537-10,71205-0537-20,71205-0537-21,71205-0537-30,71205-0537-60,71205-0537-90,00009-3475-03,00009-3475-23,00009-3475-
01,00009-3475-22,00046-1104-51,00046-1104-91,00046-1104-81,00143-9738-10,00143-9739-01,00143-9739-05,00143-9739-10,00143-9738-05,00143-9738-01,00143-9740-01,00143-9740-10,00078-0916-
61,00078-0923-61

© 2023 Bradley CJ, et al. *JAMA Health Forum.*

eTable 1. Likelihood of radiation therapy within 12 months following last surgery depending on information source, CCCR-APCD, 2012-2017, N=3219

	CCCR only, 12-month[1]	APCD only, 12-month[1]	Either APCD or CCCR
**Insurance**			
Private	Reference	Reference	Reference
Medicaid	-0.04** (-0.08, -0.01)	0.01 (-0.03, 0.04)	0.01 (-0.03, 0.04)
**Age Category**			
<50	Reference	Reference	Reference
50-63	0.16*** (0.13, 0.20)	0.10*** (0.06, 0.13)	0.11*** (0.08, 0.15)
**Race/Ethnicity Category**			
White Non-Hispanic	Reference	Reference	Reference
Hispanic	0.03 (-0.02, 0.08)	0.03 (-0.02, 0.07)	0.030 (-0.02, 0.08)
Black	0.01 (-0.08, 0.09)	0.01 (-0.07, 0.10)	0.031 (-0.05, 0.11)
Other/Unknown	0.06 (-0.03, 0.14)	0.03 (-0.05, 0.11)	0.029 (-0.05, 0.10)
**Rural residency**			
No	Reference	Reference	Reference
Yes	-0.03 (-0.09, 0.02)	-0.027 (-0.07, 0.03)	-0.03 (-0.08, 0.02)
Missing	-0.00 (-0.08, 0.07)	0.01 (-0.06, 0.08)	0.01 (-0.06, 0.08)
**SEER Summary Stage**			
Localized	Reference	Reference	Reference
Regional	0.08*** (0.04, 0.11)	0.20*** (0.16, 0.23)	0.21*** (0.18, 0.24)
**Reporting Source**			
Inpatient or hospital	Reference	Reference	Reference
Outpatient	0.01 (-0.17, 0.18)	0.075 (-0.09, 0.22)	0.08 (-0.07, 0.23)

CCCR= Colorado Central Cancer Registry; APCD=All Payer Claims Database; N/A=not applicable. Logistic regression with predicted margins reported. All women are continuously enrolled in Medicaid or an APCD plan. Year of diagnosis included, but coefficients are not reported. [1] Radiation treatment was defined as patients who received radiation therapy within 12 months following breast cancer surgery (biopsy except fine needle, breast conserving surgery, lumpectomy, lymphadenectomy, and sentinel lymphadenectomy included) from CCCR data or APCD claims data. Statistical significance noted as **p<0.05, ***p<0.01.

© 2023 Bradley CJ, et al. *JAMA Health Forum.*

eTable 2. Likelihood of hormonal therapy within 12 months following last surgery depending on information source, CCCR-APCD, 2012-2017, N=2690

	CCCR only, 12-month[1]	APCD only, 12-month[1]	Either APCD or CCCR
**Insurance**			
Private	Reference	Reference	Reference
Medicaid	-0.10*** (-0.14, -0.07)	-0.01 (-0.03, 0.02)	-0.01 (-0.03, 0.01)
**Age Category**			
<50	Reference	Reference	Reference
50-64	0.03* (-0.00, 0.07)	-0.01 (-0.03, 0.01)	0.00 (-0.01, 0.02)
**Race/Ethnicity Category**			
White Non-Hispanic	Reference	Reference	Reference
Hispanic	0.01 (-0.04, 0.06)	0.00 (-0.02, 0.03)	0.02 (-0.00, 0.03)
Black	0.03 (-0.05, 0.12)	0.00 (-0.048, 0.05)	-0.00 (-0.05, 0.04)
Other/Unknown	0.00 (-0.08, 0.09)	0.00 (-0.04, 0.05)	-0.01 (-0.05, 0.03)
**Rural residency**			
No	Reference	Reference	Reference
Yes	-0.11*** (-0.16, -0.05)	-0.01 (-0.04, 0.02)	-0.01 (-0.03, 0.02)
Missing	-0.05 (-0.12, 0.03)	0.00 (-0.03, 0.04)	0.00 (-0.03, 0.03)
**SEER Summary Stage**			
Localized	Reference	Reference	Reference
Regional	-0.08*** (-0.11, -0.04)	0.03*** (0.01, 0.05)	0.02*** (0.01, 0.04)
**Reporting Source**			
Inpatient or hospital	Reference	Reference	Reference
Outpatient	0.02 (-0.16, 0.21)	-0.10 (-0.25, 0.05)	-0.033 (-0.14, 0.07)

CCCR= Colorado Central Cancer Registry; APCD=All Payer Claims Database; N/A=not applicable. Logistic regression with predicted margins reported. All women are continuously enrolled in Medicaid or an APCD plan. Year of diagnosis included, but coefficients are not reported. [1] Hormonal therapy was defined as patients who received hormonal therapy within 12 months following breast cancer surgery (biopsy except fine needle biopsy, mastectomy, breast conserving surgery, lumpectomy, lymphadenectomy, and sentinel lymphadenectomy included) from CCCR data or APCD claims data. Statistical significance noted as *p<0.10, **p<0.05, ***p<0.01.

© 2023 Bradley CJ, et al. *JAMA Health Forum.*

eTable 3. Likelihood of radiation therapy within 9 months following last surgery depending on information source, CCCR-APCD, 2012-2017, N=3138

	**CCCR only, 9-month[1]**	**APCD only, 9-month[1]**	**Either APCD or CCCR**
**Insurance**			
Private	Reference	Reference	Reference
Medicaid	-0.03* (-0.07, 0.00)	0.01 (-0.03, 0.04)	0.01 (-0.03, 0.05)
**Age Category**			
<50	Reference	Reference	Reference
50-64	0.17*** (0.14, 0.21)	0.12*** (0.09, 0.16)	0.14*** (0.10, 0.17)
**Race/Ethnicity Category**			
Black non-Hispanic	-0.02 (-0.11, 0.06)	-0.01 (-0.10, 0.07)	0.01 (-0.08, 0.09)
Hispanic	0.02 (-0.03, 0.07)	0.00 (-0.05, 0.05)	0.01 (-0.03, 0.06)
White non-Hispanic	Reference	Reference	Reference
Other/Unknown	0.05 (-0.04, 0.13)	0.01 (-0.07, 0.09)	0.02 (-0.06, 0.09)
**Rural residency**			
No	Reference	Reference	Reference
Yes	-0.06 (-0.13, 0.02)	-0.07* (-0.14, 0.00)	-0.06* (-0.14, 0.01)
**SEER Summary Stage**			
Localized	Reference	Reference	Reference
Regional	0.06*** (0.02, 0.09)	0.18*** (0.15, 0.21)	0.19*** (0.16, 0.22)
**Reporting Source**			
Inpatient or hospital	Reference	Reference	Reference
Outpatient	-0.09 (-0.27, 0.10)	0.05 (-0.13, 0.23)	0.03 (-0.15, 0.21)
**Poverty Level[2]**			
Quantile 1	Reference	Reference	Reference
Quantile 2	-0.02 (-0.06, 0.03)	-0.01 (-0.06, 0.03)	-0.01 (-0.05, 0.04)
Quantile 3	-0.04 (-0.09, 0.01)	-0.02 (-0.07, 0.03)	-0.03 (-0.07, 0.02)
Quantile 4 (highest)	-0.03 (-0.08, 0.03)	0.00 (-0.05, 0.05)	-0.01 (-0.06, 0.04)
**Rural hospital/provider[3]**			
Urban	Reference	Reference	Reference
Rural	0.04 (-0.04, 0.12)	0.07** (0.00, 0.14)	0.06* (0.00, 0.13)

CCCR= Colorado Central Cancer Registry; APCD=All Payer Claims Database; N/A=not applicable. Logistic regression with predicted margins reported; 95% confidence intervals in parentheses. All women are continuously enrolled in Medicaid or an APCD plan. Year of diagnosis included, but coefficients are not reported. The sample of this analysis excluded those who were missing census tract poverty information (N=199) and those who were missing hospital/provider rural information from APCD (N=55). Footnotes to Table 1 apply.

[1]Radiation treatment was defined as patients who received radiation therapy within 9 months following breast cancer surgery (biopsy except fine needle, breast conserving surgery, lumpectomy, lymphadenectomy and sentinel lymphadenectomy included) from CCCR data or APCD claims data. Statistical significance noted as **p<0.05, ***p<0.01.

[2] Census Tract variable from American Community Survey (ACS) 5-Year data Income and Poverty for family poverty status (below poverty level) in the past 12 months.

[3] We defined the indicator of rural hospital or provider as if a patient had surgery in a rural hospital during the 3 months period or if a patient had a visit in a rural provider for radiation therapy.

© 2023 Bradley CJ, et al. *JAMA Health Forum.*

eTable 4. Likelihood of hormonal therapy within 9 months following last surgery depending on information source, CCCR-APCD, 2012-2017, N=2619

	CCCR only, 9-month[1]	APCD only, 9-month[1]	Either APCD or CCCR
**Insurance**			
Private	Reference	Reference	Reference
Medicaid	-0.08*** (-0.12, -0.04)	0.00 (-0.03, 0.02)	-0.01 (-0.03, 0.01)
**Age Category**			
<50	Reference	Reference	Reference
50-64	0.05** (0.01, 0.08)	-0.01 (-0.03, 0.01)	0.00 (-0.01, 0.02)
**Race/Ethnicity Category**			
Black non-Hispanic	0.04 (-0.05, 0.13)	0.03 (-0.02, 0.07)	0.01 (-0.04, 0.05)
Hispanic	0.01 (-0.04, 0.06)	0.02 (-0.01, 0.04)	0.02* (0.00, 0.04)
White non-Hispanic	Reference	Reference	Reference
Other/Unknown	0.00 (-0.09, 0.08)	0.01 (-0.03, 0.06)	0.01 (-0.03, 0.05)
**Rural residency**			
No	Reference	Reference	Reference
Yes	-0.06 (-0.13, 0.02)	-0.07** (-0.14, -0.01)	-0.06** (-0.12, -0.01)
**SEER Summary Stage**			
Localized	Reference	Reference	Reference
Regional	-0.13*** (-0.16, -0.09)	0.03*** (0.01, 0.05)	0.01 (-0.01, 0.03)
**Reporting Source**			
Inpatient or hospital	Reference	Reference	Reference
Outpatient	0.01 (-0.19, 0.22)	-0.13 (-0.31, 0.05)	-0.05 (-0.18, 0.08)
**Poverty Level[2]**			
Quantile 1	Reference	Reference	Reference
Quantile 2	-0.04 (-0.09, 0.01)	-0.01 (-0.04, 0.02)	0.00 (-0.03, 0.02)
Quantile 3	-0.04 (-0.09, 0.01)	0.01 (-0.02, 0.03)	0.00 (-0.03, 0.02)
Quantile 4 (highest)	-0.04 (-0.10, 0.01)	0.01 (-0.01, 0.04)	0.00 (-0.02, 0.03)
**Rural hospital/provider[3]**			
Urban	Reference	Reference	Reference
Rural	-0.06 (-0.13, 0.02)	0.05*** (0.03, 0.07)	0.03*** (0.01, 0.05)

CCCR= Colorado Central Cancer Registry; APCD=All Payer Claims Database; N/A=not applicable. Logistic regression with predicted margins reported; 95% confidence intervals in parentheses. All women are continuously enrolled in Medicaid or an APCD plan. Year of diagnosis included, but coefficients are not reported. The sample of this analysis excluded those who were missing census tract poverty information (N=162) and those who were missing hospital/provider rural information from APCD (N=42). Footnotes to Table 1 apply.

[1]Hormonal therapy was defined as patients who received hormonal therapy within 9 months following breast cancer surgery (biopsy except fine needle biopsy, mastectomy, breast conserving surgery, lumpectomy, lymphadenectomy and sentinel lymphadenectomy included) from CCCR data or APCD claims data. Statistical significance noted as ***p<0.01.

[2] Census Tract variable from American Community Survey (ACS) 5-Year data Income and Poverty for family poverty status (below poverty level) in the past 12 months.

[3] We defined the indicator of rural hospital or provider as if a patient had surgery in a rural hospital during the 3 months period or if a patient had a visit in a rural provider for hormonal therapy.

© 2023 Bradley CJ, et al. *JAMA Health Forum.*
