# Supplementary material for: Treatment Disparities in Radiation and Hormone Therapy Among Women Covered by Medicaid vs Private Insurance in Cancer Registry and Claims Data
Source: JAMA Health Forum. 2023 May 5;4(5):e230673. doi: 10.1001/jamahealthforum.2023.0673 (PMC10163382; doi:10.1001/jamahealthforum.2023.0673)
Supplement: Supplement 2. — Data Sharing Statement [file jamahealthforum-e230673-s002.pdf]

Data Sharing Statement

Bradley. Treatment Disparities in Radiation and Hormone Therapy Among Women Covered by Medicaid vs Private Insurance in Cancer Registry and Claims Data. *JAMA Health Forum*. Published May 05, 2023. doi:10.1001/jamahealthforum.2023.0673

Data
Data available: Yes
Data types: Deidentified participant data
How to access data: Data are available under the conditions of the Data Use Agreement.
When available: With publication

Supporting Documents
Document types: None

Additional Information
Who can access the data: Data may be shared under the conditions of the data use agreement with the Center for Improving Value in Health Care.
Types of analyses: for any purpose
Mechanisms of data availability: Data may be shared under the conditions of the data use agreement with the Center for Improving Value in Health Care.
